# Mechanistic Pathways and Product Selectivity in Pyrolysis of PE, PP and PVC: A Foundation for Applied Chemistry in Europe

**DOI:** 10.3390/molecules31020202

**Published:** 2026-01-06

**Authors:** Tim Tetičkovič, Dušan Klinar, Klavdija Rižnar, Darja Pečar

**Affiliations:** 1 Scientific Research Centre Bistra Ptuj, Slovenski Trg 6, 2250 Ptuj, Slovenia; dusan.klinar@bistra.si (D.K.); klavdija.riznar@bistra.si (K.R.); 2 Faculty of Chemistry and Chemical Engineering, University of Maribor, Smetanova Ulica 17, 2000 Maribor, Slovenia; darja.pecar@um.si

**Keywords:** pyrolysis, catalytic pyrolysis, reaction mechanism, product selectivity, vapor residence time, initiators, light olefins/BTX

## Abstract

Plastic streams dominated by polyethylene (PE) including PE HD/MD (High Density/Medium Density) and PE LD/LLD (Low Density/Linear Low Density), polypropylene (PP), and polyvinyl chloride (PVC) across Europe demand a design framework that links synthesis with end of life reactivity, supporting circular economic goals and European Union waste management targets. This work integrates polymerization derived chain architecture and depolymerization mechanisms to guide selective valorization of commercial plastic wastes in the European context. Catalytic topologies such as Bronsted or Lewis acidity, framework aluminum siting, micro and mesoporosity, initiators, and strategies for process termination are evaluated under relevant variables including temperature, heating rate, vapor residence time, and pressure as encountered in industrial practice throughout Europe. The analysis demonstrates that polymer chain architecture constrains reaction pathways and attainable product profiles, while additives, catalyst residues, and contaminants in real waste streams can shift radical populations and observed selectivity under otherwise similar operating windows. For example, strong Bronsted acidity and shape selective micropores favor the formation of C_2_ to C_4_ olefins and Benzene, Toluene, and Xylene (BTX) aromatics, while weaker acidity and hierarchical porosity help preserve chain length, resulting in paraffinic oils and waxes. Increasing mesopore content shortens contact times and limits undesired secondary cracking. The use of suitable initiators lowers the energy threshold and broadens processing options, whereas diffusion management and surface passivation help reduce catalyst deactivation. In the case of PVC, continuous hydrogen chloride removal and the use of basic or redox co catalysts or ionic liquids reduce the dehydrochlorination temperature and improve fraction purity. Staged dechlorination followed by subsequent residue cracking is essential to obtain high quality output and prevent the release of harmful by products within European Union approved processes. Framing process design as a sequence that connects chain architecture, degradation chemistry, and operating windows supports mechanistically informed selection of catalysts, severity, and residence time, while recognizing that reported selectivity varies strongly with reactor configuration and feed heterogeneity and that focused comparative studies are required to validate quantitative structure to selectivity links. In European post consumer sorting chains, PS and PC are frequently handled as separate fractions or appear in residues with distinct processing routes, therefore they are not included in the polymer set analyzed here. Polystyrene and polycarbonate are outside the scope of this review because they are commonly handled as separate fractions and are typically optimized toward different product slates than the gas, oil, and wax focused pathways emphasized here.

## 1. Introduction

Diverting plastic waste away from landfills is no longer just a matter of space and leachate control; it is an opportunity to recover carbon as useful energy carriers and chemicals via thermal and catalytic valorization [1,2,3]. In this manuscript, the term polyolefins is used in the chemical sense for olefin derived polymers, including PE, PP, and PVC. For clarity in process design, PE and PP are often discussed together, whereas PVC is discussed in dedicated parts because chlorine driven dehydrochlorination introduces distinct constraints on reactor operation, corrosion, and product purification. Across these polymers, chain architecture formed during polymerization provides a baseline for radical formation and scission statistics after melting, while processing history, ageing, additives, impurities, and operating conditions can widen and shift the observed kinetics and product distributions [1,2,3,4,5]. The aim of this review is to provide a defensible process design bridge for PE including PE HD MD and PE LD LLD, PP, and PVC by linking polymerization derived chain architecture to dominant thermolysis pathways, and by showing how operating variables and intervention layers (catalysts, initiators, and halogen management) shift the product window between gas, oil, wax, and aromatics. Because post consumer feeds differ in additive packages, catalyst residues, and contamination levels, the structure and selectivity discussed in this review are interpreted as mechanistic tendencies within reported operating windows, not as universal quantitative correlations. In this review, structure selectivity links are stated only as directional trends within stated operating windows, and Tables 2 and 3 report the minimum basis conditions (reactor mode, temperature program, heating rate, vapor residence time, atmosphere or pressure, and for catalysts the catalyst to polymer ratio and contact mode) required for a defensible comparison.

This review focuses on the dominant olefin derived polymer streams relevant for thermal recycling of European post consumer plastics, specifically PE including PE LD and PE LLD, PE HD and PE MD, PP. Polystyrene (PS) and polycarbonate (PC) are not treated in detail in this work because their pyrolysis pathways and target products differ from the oil and wax oriented product windows emphasized here, and because they are typically managed as separate fractions in practical sorting and processing routes. LLDPE is addressed within the PE LD family because its short chain branching architecture and packaging relevance place it within the same mechanistic and process design envelope for pyrolysis severity and product distribution. Strategic catalytic interventions—hydrogenolysis, metathesis, and acid site mediated cracking on micro/mesoporous frameworks—translate that mechanistic control into targeted product slates while minimizing waste formation [1,2,3].

For HDPE, LDPE and PP, well resolved kinetic and mechanistic studies converge on random C–C scission, β-scission, hydrogen abstraction, and backbiting as the primary routes that set the baseline gas vs. condensable yields [6,7]. Process severity and heat-flux history strongly modulate light olefin formation; under high heating rates, pressure and heating rate redistribute diene/alkene/alkane balances, while parametric datasets with advanced analytics clarify how temperature and residence time steer primary volatiles without prolonged cracking [8,9]. Mixed virgin and waste PP and PE LD/LLD systems exhibit lowered apparent barriers, enabling comparable conversions at milder temperatures—an operational advantage for waste streams [10]. Once acids are introduced, shape selectivity compresses distributions toward gasoline/BTX cuts over Hydrogen—form Zeolite Socony Mobil-5 (HZSM-5), whereas weaker acidity and larger pores, coupled with rapid quenching, preserve longer chains conducive to wax/oil fractions [11]. PP’s tertiary centers accentuate formation of C_1_–C_4_ and gasoline range intermediates at moderate severities; temperature programmed profiles delineate the window to pivot between off gas for combined heat and power (CHP) and liquids for upgrading [12].

PVC requires a sequenced flowsheet. The first stage is controlled dehydrochlorination, governed by zipper type autocatalysis captured by classical kinetics and supported by evidence for parallel unimolecular and radical pathways; a modern two step kinetic picture aligns with these fundamentals and explicitly links the structure of the dehydrochlorinated residue to subsequent reactivity [13,14,15]. Selectivity improves when HCl is continuously removed. Additives and processing strategies that curtail HCl activity slow the autocatalytic loop and redirect pathways [16]. Metal chlorides/oxides lower the onset temperature for dehydrochlorination and can suppress aromatization from polyenes, enabling cleaner gases and residues for downstream conversion [17,18]. When aromatics form, they arise via intramolecular cyclizations of conjugated sequences—a mechanistic insight that justifies short residence and the use of mild basic/redox cocatalysts downstream to limit condensation [19]. Halogen–metal interactions matter operationally: Cu-based species markedly perturb HCl/volatile organic compound (VOC) release profiles, underscoring the need for halogen guards and compatible metallurgy [20]. At the molecular scale, defect-assisted chlorine migration and Thermogravimetric—Fourier Transform Infrared Spectroscopy (TG-FTIR) diagnostics on cable grades reinforce an analytics led two stage design (dechlorination → residue cracking) and help place capture media and catalysts effectively [21,22].

Catalyst microstructure, especially the balance of Brønsted and Lewis acidity, framework Al siting, and pore architecture, strongly influences how polymer derived intermediates traverse these degradation networks under a given temperature and residence time regime. Hierarchical ZSM-5 families and tuned acidity/alkalinity increase light olefin and aromatic yields while suppressing coke by mitigating secondary hydrogen transfer and cyclization in tight micropores [23,24,25]. Micropore topology dictates where coke nucleates and grows, linking void geometry to deactivation kinetics and selectivity loss [26]. Precise placement of framework Al can tilt pathways toward primary β-scission over secondary oligomerization, boosting olefin formation from LDPE/PP feeds [27].

Mechanistically, acidity aware molecular kinetic models and first-principles microkinetics illuminate when monomolecular cracking, bimolecular hydride transfer, or aromatization dominate, and how temperature or acid site density toggles among these regimes [28,29]. Zeolite confined β-scission barriers depend jointly on hydrocarbon structure and pore confinement, providing predictive handles to match feed microstructure (branching, unsaturation) with catalyst topology [30].

Operational levers couple with microstructure to steer outcomes. CO_2_ cofeeds promote dehydrogenation and shift equilibria toward aromatics over mesoporous HZSM-5 and Ga/ZSM-5, reducing coproduced H_2_ [31]. In PP/PE upgrading, mesopore enrichment shortens contact times and curbs secondary reactions, raising propylene/olefin selectivity without deep cracking [32]. Phase aware residence time control and staged/co feed strategies modulate pool chemistry, enabling concurrent formation of ethylene/propylene and para-xylene on a single catalyst bed when architecture and conditions are cooptimized [33,34].

**Figure 1:**
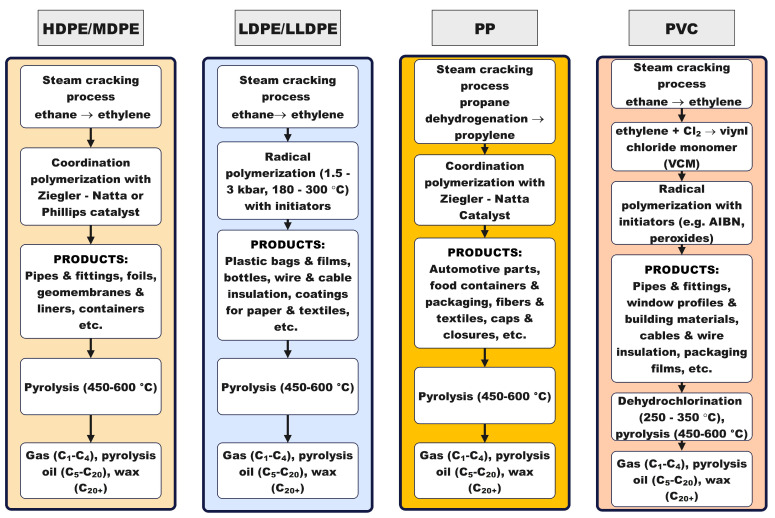
Overview of the pathway from raw materials to pyrolysis for PE HD/MD, PE LD/LLD, PP, and PVC. Monomer supply (ethylene, propylene, VCM) and polymerization mode (coordination or radical) set the chain microstructures (branching, tertiary C, C–Cl) that influences baseline radical formation and scission tendencies after melting. At the end of life, fractions unsuitable for mechanical recycling are routed to pyrolysis. Dominant degradation modes are random/β-scission (HDPE), random scission with H-abstraction/backbiting (LDPE), β-scission (PP), and zipper dehydrochlorination to HCl and a polyene residue (PVC). Operating variables (temperature, heating rate, residence time, rapid quench) and optional catalysts/initiators (e.g., zeolites, metals, peroxides) tune product windows from gases to oils/waxes; for PVC, staged dechlorination with HCl capture precedes cracking of the residue.

Stability hinges on diffusion management and acid site tailoring. Micropore-diffusion control and hierarchical/core–shell architectures delay pore mouth blockage and lower coke yield; phosphorus modification or external surface passivation further balances acidity to sustain activity at high conversion [25,35,36]. Pairing mesoporous domains in bifunctional systems limits methane and other light gas losses during hydrogenolysis/hydrocracking, extending time on stream while biasing liquids over gases [37]. Recent demonstrations with H-form MFI-type zeolite (HMFI) catalysts show that PP can be converted to light olefins even below classical pyrolytic temperatures when carbocation chemistry and external surface sites are leveraged, broadening the operable window for selective, energylean upgrading [38]. Figure 1 synthesizes this structure, mechanism and operation logic: starting from monomer supply and polymerization mode (Ziegler–Natta or radical) that largely defines chain architecture of PE HD/MD, PE LD/LLD, PP, and PVC, it maps the dominant thermolysis routes (random/β-scission, H-abstraction/backbiting; zipper dehydrochlorination for PVC), the key operating levers (temperature, heating rate, residence time, rapid quench), and the role of optional catalysts/initiators and HCl management (for PVC) in steering outcomes from light gases to paraffinic oils/waxes.

A mechanistic viewpoint converts “non mechanistic operating window selection” into design: it identifies which bonds break first, which intermediates dominate, and where to intervene with catalysts (acid strength, metal function, topology), initiators (to set onset and radical populations), and suppressors/rapid quench (to limit secondary cracking and preserve chain length). That same insight maps operating envelopes, such as heating rate ramps and final temperatures, that bias gases toward cogeneration rather than paraffinic waxes/oils suitable for candles and heat storage materials (e.g., paraffins)—Phase Change Materials (PCMs)—turning landfilled liabilities into controllable product slates with defensible process conditions.

## 2. Polymerization—Derived Microstructure of HDPE, LDPE, PP, and PVC

In this review, “microstructure” is used in the molecular sense (chain architecture), i.e., branching (SCB/LCB), stereoregularity/tacticity, defect motifs (e.g., tertiary C sites, labile chlorides, head-to-head units), and molecular weight distribution. We refer to semicrystalline morphology (crystallinity, lamellae, amorphous/crystalline domains) separately as “morphology”, because it depends not only on polymerization but also on processing and thermal history (cooling rate, ageing, etc.). Since PE/PP pyrolysis occurs after melting, morphology is not treated as a primary mechanistic control variable in the molten degradation regime; the focus in Section 2 is the chain architecture that remains present in the melt and governs radical formation and scission statistics.

For clarity, polyethylene is treated using HDPE and LDPE as bounding chain architectures, while LLDPE is discussed within the PE LD family for packaging relevant waste streams.

Industrial production routes differ across the polymer set treated in this review. HDPE and PP are manufactured predominantly by coordination polymerization using Ziegler Natta and metallocene catalyst systems, which is why Section 2.1 focuses on coordination insertion control of chain architecture. LDPE is treated through high pressure free radical polymerization and PVC through radical routes used in industrial practice, which is why Section 2.2 is written in a radical polymerization context. Other polymerization modes exist for some monomers, including ionic routes, but they are not required for the specific PE HD/MD, PE LD/LLD, PP, and PVC scope of this review or for the structure to degradation argument developed later.

### 2.1. Coordination Polymerization Mechanism of HDPE/MPDE and PP

Coordination polymerization initiates by forming an active metal-alkyl complex at a Group IV transition metal site, typically involving titanium or zirconium as the central metal. This species is generated in situ by reacting a stable precatalyst with an alkylaluminum co-catalyst, such as triethylaluminum and methylaluminoxane (e.g., Et_3_Al, MAO), which simultaneously alkylates the metal and removes ligands, creating a vacant coordination site. This vacancy allows the olefin to bind via π-complexation, where electron donation and back-donation between the monomer and the metal stabilize the complex [39,40,41]. In titanium-based systems, this interaction facilitates 1,2-insertion of the monomer into the metal–carbon bond, forming the first covalent link in the polymer chain and converting the site into an active center for propagation. Initially, Ti(IV) alkyl cations were proposed as the active species, but recent studies show that Ti(III) alkyls, formed by partial reduction with the co-catalyst, often dominate. These Ti(III) species are more effective due to their unpaired electron, which enhances monomer coordination and insertion [39]. In copolymerizations (e.g., ethylene–propylene), each monomer follows the same insertion pathway, though differences in reactivity lead to a mix of random and block segments [42]. Activation energies for initiation are moderate, ranging from 33 to 67 kJ mol^−1^, depending on the catalyst type. Homogeneous systems generally show lower barriers, supporting efficient initiation under mild conditions, though high co catalyst concentrations may still promote side reactions [43,44,45,46].

In the coordination polymerization of HDPE, chain propagation proceeds via repeated insertion of ethylene monomers into a growing metal–carbon bond at an active center such as Ti–C or Zr–C. This occurs through a classical migratory insertion mechanism, which begins with π-complexation of ethylene to a vacant site on the transition metal, followed by insertion into the metal–alkyl bond [46]. In Ziegler–Natta systems, propagation is highly stereoselective, leading to linear HDPE with low branching. Metallocene catalysts, such as zirconocene complexes, allow more precise control over polymer architecture [47]. Computational studies show that while the first few insertions face slightly increasing energy barriers due to steric hindrance and agostic interactions, these barriers later decrease, stabilizing the propagation process [48].

**Figure 2:**
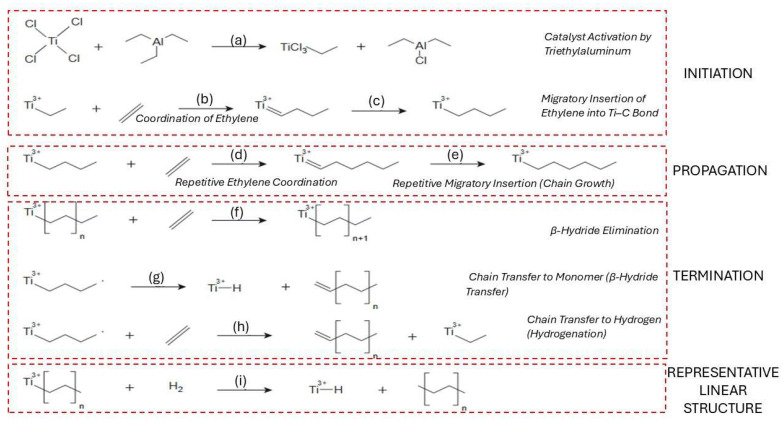
Proposed reaction mechanism for the coordination polymerization of ethylene to HDPE using a TiCl_4_/Al(C_2_H_5_)_3_ Ziegler–Natta catalytic system. (**a**) Activation of the TiCl_4_ catalyst by triethylaluminum, forming an active titanium–ethyl species, (**b**) π-coordination of ethylene to the Ti^3+^ center, (**c**) migratory insertion of ethylene into the Ti–C bond, extending the chain by two carbon atoms, (**d**) coordination of another ethylene molecule to the titanium center, (**e**) repeated migratory insertions of ethylene during chain propagation, (**f**) chain termination by β-hydride elimination forming a terminal double bond, (**g**) chain transfer to monomer via β-hydride transfer, producing a saturated chain and regenerating a Ti–ethyl site, (**h**) chain transfer to hydrogen (hydrogenation), terminating the chain with a fully saturated alkyl end, and (**i**) formation of the final linear HDPE chain structure, which is highly crystalline due to minimal branching.

The coordination of ethylene to the active site involves π-complexation, wherein the ethylene molecule donates electron density to the empty d-orbitals of the titanium center, forming a non-covalent π-complex [39,49]. This coordination orients the monomer for the subsequent insertion step but does not yet form a covalent bond. This step, along with the preceding catalyst activation, is illustrated in Figure 2a,b.

The migratory insertion of the coordinated ethylene into the Ti–C bond marks the beginning of chain growth, also known as chain initiation (Figure 2c) [50]. This step results in the formation of a new Ti–CH_2_–CH_2_–R species, where R denotes the rest of the growing polymer chain. Once the first insertion has occurred, the catalyst–polymer complex is fully activated for propagation [51]. Propagation in the coordination polymerization of HDPE involves the repeated migratory insertion of ethylene monomers into the growing metal–carbon bond, as shown in Figure 2d [52]. Each propagation step requires a fresh coordination of ethylene, followed by insertion into the Ti–C bond at the active site, typically a titanium–alkyl species. This process is highly stereoselective and normally yields linear polyethylene with minimal branching when Ziegler–Natta or metallocene catalysts are used [39,53].

Chain termination occurs through several competing mechanisms, the most common being β-hydride elimination (BHE), β-hydride transfer to monomer (BHT), and hydrogenolysis (chain transfer to hydrogen). In β-hydride elimination, a β-hydrogen from the growing polymer chain is transferred to the metal, resulting in the formation of a vinyl-terminated polymer and a metal–hydride species (Figure 2e). This pathway becomes more dominant at low monomer concentration and is generally less energetically favored than BHT [54]. β-Hydride transfer to the monomer, shown in Figure 2f, involves transferring a β-hydrogen to a nearby ethylene molecule rather than to the metal center, producing a saturated chain end and regenerating the active site. This route is often preferred due to its lower activation energy and ability to maintain polymerization activity, especially under ethylene-rich conditions [55,56]. Finally, chain transfer to hydrogen, or hydrogenation, involves hydrogenolysis of the metal–carbon bond (Figure 2g). This process leads to saturated alkyl-terminated chains and is commonly used industrially to control molecular weight distribution. It is highly selective and depends on hydrogen concentration and the catalyst’s structure [57,58]. Finally, chain transfer to hydrogen, or hydrogenation, involves hydrogenolysis of the metal–carbon bond (Figure 2h). This process leads to saturated alkyl-terminated chains and is commonly used industrially to control molecular weight distribution. It is highly selective and depends on both hydrogen concentration and the catalyst’s structure [58]. The resulting linear polyethylene structure with minimal branching is represented in Figure 2i.

**Figure 3:**
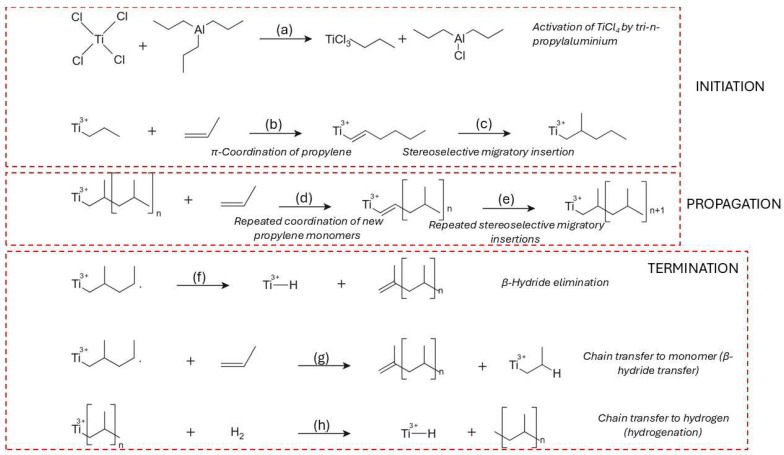
Reaction mechanism pathway of the synthesis of PP using the Ziegler-Natta catalyst TiCl_4_. (**a**) The catalyst activation or reduction of Ti(IV) to Ti(III) and alkylization of the transition metal center, and formation of the Ti^3+^-ethyl complex; (**b**) monomer coordination or π complex formation; (**c**) migratory insertion or chain initiation; (**d**) chain propagation (repeated migratory insertions); (**e**) chain termination—β hydride elimination; (**f**) chain termination—β-hydride transfer to monomer (**g**) chain termination—hydrogenation or chain transfer to hydrogen; (**h**) chain transfer to hydrogen (hydrogenation).

In the coordination polymerization of PP, the process initiates with catalyst activation identical to HDPE, involving the formation of a Ti(III)–ethyl complex on MgCl_2_ after alkylation by AlEt_3_ (Figure 3a) [59]. Propylene coordinates to the active site via its C=C double bond, forming a π-complex with a specific facial orientation, governed by the stereoelectronic environment of the metal center (Figure 3b). This orientation dictates the regio- and stereochemistry of insertion, which occurs via migratory insertion of the CH_2_ group into the Ti–C bond (Figure 3c) [60]. The resulting growing chain maintains a defined stereoregularity—either isotactic, syndiotactic, or atactic—depending on the symmetry and ligand design of the catalyst system [61]. Electron donors, both internal (e.g., diisobutyl phthalate) and external (e.g., silane-based modifiers), fine-tune this environment by altering the electron density at the titanium center and the π-complex coordination geometry [62,63].

The propagation of PP chains continues via successive insertions, each guided by steric and electronic effects that enforce specific tacticity. C_2_-symmetric zirconocene catalysts are known to promote high isotacticity through chiral induction and enantiomorphic site control [64]. Kinetic studies have shown that the first propylene insertion is stereodetermining, while subsequent insertions propagate the same configuration unless interrupted by chain transfer or site deactivation (Figure 3d,e) [65]. Metallocenes and advanced Ziegler–Natta catalysts can achieve turnover frequencies ranging from 0.1 to 1 monomer per active site per second under optimized conditions [66].

Termination mechanisms in PP polymerization are more diverse due to the additional methyl group on the monomer, which permits β-methyl elimination in addition to β-hydride elimination. In β-methyl elimination, the methyl group is removed from the β-position and transferred to the metal center, forming allylic or branched end-groups [67]. β-Hydride elimination, the more common route, leads to vinyl-terminated PP chains and Ti–H species (Figure 3f), whereas β-hydride transfer to monomer results in a new Ti–alkyl center and a terminally unsaturated chain (Figure 3g) [68,69]. Octahedral zirconium complexes with carefully tuned ligand geometries demonstrate comparable precision: while one catalyst may yield site-controlled isotactic chains, a structurally similar complex may produce syndiotactic polymers under chain-end control, emphasizing the sensitivity of stereochemical outcome to ligand design [70]. The migratory insertion mechanism becomes essential in specific catalyst systems, particularly those based on C_2_-symmetric s-bis(indenyl) {SBI}-type zirconocenes. These catalysts direct monomer coordination to the sterically crowded site, favoring chain migration and high isotacticity. Thermochemical modeling of the first few insertion steps correlates well with experimental pentad distributions, confirming that migratory insertion mechanisms dominate in these systems and are necessary for achieving highly isoselective propagation [71].

Chain transfer to hydrogen is especially significant industrially, where molecular hydrogen is used to produce PP with reduced molecular weight and improved processability (Figure 3h) [57,58]. This pathway involves the cleavage of the metal–carbon bond by hydrogen, forming a saturated polymer and a new Ti–H center, which can reinitiate polymerization or become deactivated depending on the system [72].

Computational studies have further refined these mechanistic pathways, revealing two dominant transition states in β-hydride transfer: Syn Transition State (TSA), which favors direct metal–hydrogen bonding and is common in less sterically hindered environments, and Anti Transition State (TSC), more prominent in bulkier catalysts where propagation is sterically hindered and termination is favored [56,73]. Subtle changes in ligand geometry, donor type, and monomer concentration govern the selection between these routes. These findings demonstrate the crucial role of catalyst design and polymerization environment in determining the final properties of PP.

Overall, coordination polymerization of HDPE and PP follows a fundamentally similar mechanism driven by metal-catalyzed coordination–insertion, but their differences in monomer structure led to distinct stereochemical pathways, termination behavior, and catalyst requirements. The design of catalyst ligands, use of electron donors, and operational parameters such as hydrogen pressure and monomer concentration together allow precise control of polymer microstructure, molecular weight distribution, and end-group functionality. These parameters must be finely tuned to meet specific industrial requirements, particularly for the sustainable and efficient synthesis of HDPE, LDPE and PP under modern green chemistry constraints.

### 2.2. Free Radical Polymerization of LDPE and PVC

**Figure 4:**
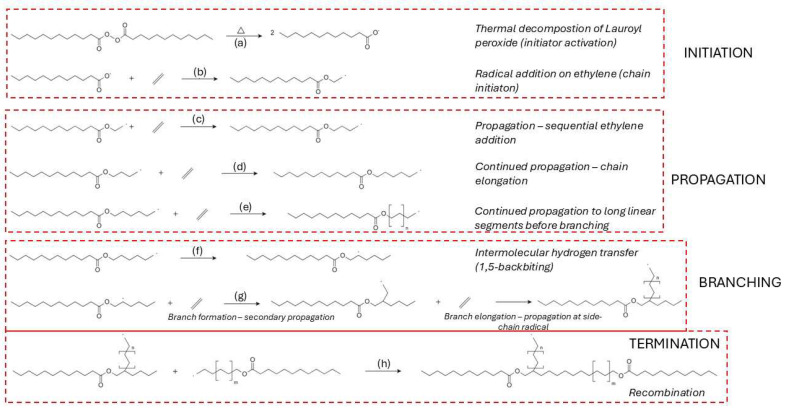
Proposed free radical polymerization mechanism for the formation of LDPE using lauroyl peroxide as initiator. (**a**) Decomposition of lauroyl peroxide yields two primary radicals, (**b**) radical addition to ethylene initiates chain growth, (**c**) propagation by sequential ethylene additions, (**d**) continued linear propagation via repetitive monomer insertions, (**e**) extended chain growth under high-pressure conditions, (**f**) intramolecular hydrogen transfer (1,5-backbiting) forms a secondary internal radical, (**g**) branch formation via ethylene addition at the side-chain radical site, with branch elongation through continued propagation from the newly formed branch point, and (**h**) termination by recombination of two radical chain ends.

The synthesis of LDPE via high-pressure free radical polymerization is initiated by the thermal decomposition of organic peroxides, which generate reactive radicals capable of initiating chain growth [74,75,76,77]. The efficiency of this initiation step is highly dependent on the peroxide’s molecular structure, decomposition kinetics, and compatibility with process parameters such as temperature, pressure, and residence time [75,78]. Among the commonly used initiators, lauroyl peroxide is frequently applied due to its predictable decomposition behavior [79]. The decomposition pathway of lauroyl peroxide is illustrated in Figure 4a, where homolytic O–O bond cleavage leads to the formation of two acyloxy radicals [79]. These radicals subsequently react with ethylene monomers to form the first macroradical species, as shown in Figure 4b [80].

Initiator structure plays a pivotal role in defining polymerization performance [81]. For instance, bi-functional peroxides produce multiple radical centres upon decomposition, enabling more rapid initiation and increased monomer conversion relative to their mono-functional counterparts [82]. This enhanced efficiency is crucial for maintaining high yields and narrow molecular weight distributions, especially under the extreme conditions characteristic of LDPE production [83]. Meanwhile, tetrafunctional initiators like JWEB50 have been shown to outperform conventional mono-functional initiators in other monomer systems, underscoring the broader utility of multifunctional peroxides in radical polymerizations [84].

The decomposition of organic peroxides typically proceeds via homolytic O–O bond cleavage, producing acyloxy, alkyl, or alkoxy radicals depending on the peroxide type [85]. These radicals differ in reactivity and thermal stability, attributes that can be fine-tuned through steric and electronic modifications to the initiator structure [86]. For example, dialkyl peroxides yield fast initiation with reduced side reactions, while hydroperoxides and ketone peroxides, though reactive, pose safety concerns and are limited to niche applications [76].

Initiator performance evolves with changes in process conditions [87]. Each peroxide displays an optimal temperature range where radical generation is maximized and undesired side reactions are minimized [88]. Beyond this range, excessive decomposition reduces radical efficiency, diminishing polymer yield despite higher initiator consumption [89]. The introduction of composite initiation strategies—such as combining azo and peroxide initiators—has been explored to balance radical reactivity and propagation control, especially in systems where fine-tuning molecular architecture is desirable [76,85,90].

Overall, initiation in LDPE polymerization hinges on precise radical generation via carefully selected peroxides [76,85,90]. By optimizing initiator structure, dosing, and thermal behavior in response to reactor conditions, industrial processes can achieve efficient chain initiation with minimal waste and maximum product control [91].

Propagation in the free radical polymerization of LDPE proceeds through the sequential addition of ethylene monomers to growing chain radicals, a process governed by a delicate balance between chain growth, intermolecular chain transfer, and intramolecular branching [92,93,94]. This propagation sequence is shown in Figure 4c–e [92]. One of the dominant branching mechanisms is 1,5-hydrogen backbiting, where a hydrogen atom is abstracted from a methylene unit five carbon atoms upstream, producing a secondary or tertiary radical that enables the formation of short-chain branches—an essential structural feature of LDPE [92]. This reaction is shown in Figure 4f, where the terminal radical folds back and abstracts a hydrogen atom from an internal CH_2_ group, generating a branched radical that can further propagate (Figure 4g) [79,89,94]. This pathway has a relatively low activation energy, 54 kJ mol^−1^, making it kinetically favorable under high-pressure industrial conditions [95].

The competing intermolecular chain transfer reactions—particularly those involving hydrogen abstraction by solvent, monomer, or polymer—exhibit higher activation energies (~100 kJ mol^−1^) and primarily regulate molecular weight [96,97]. All three kinetic processes—propagation, chain transfer, and branching—scale quadratically with ethylene fugacity, reinforcing the role of pressure [98]. Kinetic models have been developed that explicitly account for primary propagation and secondary branching mechanisms [99,100]. These simulate molecular weight and branching distributions by solving recursion equations under realistic reactor conditions [101].

Termination occurs predominantly through bimolecular reactions between macroradicals, with recombination (combination) and disproportionation as the main pathways [96]. These are exemplified in Figure 4h, where two growing macroradicals undergo recombination to form a saturated polymer chain [102]. Chain-length-dependent termination (CLDT) has been confirmed using Pulsed Laser Polymerization (PLP) and Electron Paramagnetic Resonance (EPR) techniques, showing that short radicals terminate faster than longer ones [103,104]. Activation energy for termination is also chain-length dependent: short chains exhibit Ea of 25–39 kJ mol^−1^, while longer radicals show values of 18–24 kJ mol^−1^ [105]. The selectivity between termination modes is influenced by viscosity, radical size, and temperature. Higher temperatures and lower viscosities favor disproportionation, while viscous media promote recombination [102].

Termination dynamics are modelled using MWD-based simulations and Monte Carlo approaches that account for diffusion, backbiting, and gel effects [91]. Advanced techniques such as Single Pulse-Pulsed Laser Polymerization-Electron Paramagnetic Resonance (SP-PLP-EPR) quantify propagation and backbiting effects with high resolution [106,107].

**Figure 5:**
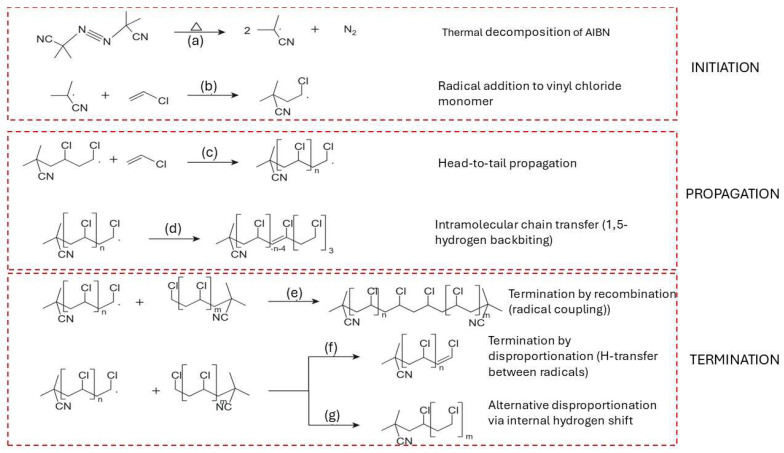
Proposed mechanism of free radical polymerization of vinyl chloride (VC) using AIBN as initiator. (**a**) Thermal decomposition of AIBN generates two 2-cyano-2-propyl radicals and nitrogen gas. (**b**) The primary radical adds to vinyl chloride, forming an initiating macroradical. (**c**) Propagation occurs via head-to-tail addition of successive vinyl chloride monomers, yielding a stereoregular –CH_2_–CHCl– polymer backbone. (**d**) Intramolecular chain transfer through 1,5-hydrogen backbiting forms a mid-chain radical, initiating branching. (**e**) Termination by recombination, where two macroradicals couple to form a saturated chain. (**f**) Termination by disproportionation via hydrogen atom transfer, producing one saturated and one unsaturated PVC chain. (**g**) An alternative disproportionation route, involving an internal hydrogen shift, yields a saturated polymer end-group.

The free radical polymerization of vinyl chloride (VC) is initiated by the thermal decomposition of azo compounds such as azobisisobutyronitrile (AIBN), which cleaves into two carbon-centered radicals [85,92,102]. This decomposition step is depicted in Figure 5a. The generated radicals add to vinyl chloride monomers, resulting in the formation of primary macroradicals as shown in Figure 5b [108]. Chain propagation proceeds predominantly through head-to-tail monomer addition, forming a linear and stereoregular structure, illustrated in Figure 5c [109]. The propagation step is highly exothermic, with activation energies at 24.9 kJ mol^−1^, depending on the tacticity and chain environment [110,111].

Intramolecular chain transfer via 1,5-hydrogen backbiting becomes significant as conversion increases, with the activation energy estimated at around 54.3 kJ mol^−1^ [94,111]. This step is visualized in Figure 5d, where the growing terminal radical abstracts a hydrogen atom from a methylene group within the chain, forming a mid-chain radical (MCR) [112,113,114]. The rate constant for the first propagation step from the branching site (BP1) is several orders of magnitude lower than for terminal propagation, although subsequent propagation steps (BP2, BP3) recover near-terminal reactivity [111,113,115].

At high monomer concentrations, chain transfer to monomer occurs via hydrogen abstraction, creating a new initiating radical and terminating the existing chain [113,116]. Although slower than propagation, this process becomes significant at elevated conversions and influences both molecular weight and the distribution of internal unsaturations [116,117]. Chain transfer to polymer results in long-chain branching and higher polydispersity, especially under bulk polymerization conditions [116].

Internal double bonds are formed through β-chlorine elimination or long-range hydrogen transfer [117]. These unsaturations behave like chain-end groups and contribute to thermal and UV instability of the polymer [118]. Stereoselective backbiting is also known to introduce tacticity-related defects, particularly at lower conversions when radicals exhibit greater conformational flexibility [119].

As polymerization proceeds toward high conversion (>85%), monomer availability in the reaction phase decreases sharply, and radical propagation slows due to increasing viscosity and reduced chain mobility [109,120]. Under these conditions, propagation shifts toward monomolecular reactions, and structural defects such as branching and internal unsaturation increase [120]. Chloroallylic end groups, commonly associated with chain termination, decrease in frequency at these stages, indicating a shift in dominant termination mechanisms [120,121].

Figure 5e–g summarizes the principal radical termination pathways relevant for radical chain reactions in general and it is included here as a conceptual reference for later degradation discussions, not as a claim that polymerization termination and pyrolysis termination are identical. In recombination (Figure 5e) [122], two macroradicals couple to form a single, saturated polymer chain, thereby neutralizing radical activity. Disproportionation (Figure 5f) entails hydrogen transfer from one radical to another, resulting in a saturated and an unsaturated chain, while a variant shift mechanism (Figure 5g) [123] yields a saturated molecule through internal stabilization. The balance between recombination and disproportionation is regulated by factors such as radical chain length, temperature, and melt viscosity [123,124]. Typically, short-chain radicals, owing to their higher diffusion rates, favor disproportionation, whereas larger, more hindered radicals promote recombination [105].

CLDT has been confirmed through experiments using pulsed laser polymerization and EPR spectroscopy [105,123]. These findings are relevant for modeling termination kinetics and predicting molecular weight distributions in industrial PVC production [109].

Chain transfer appears to play a significant role in PVC polymerization, particularly under high conversion and bulk-phase conditions. The observed number of initiator-derived end groups per chain is often below 0.4, which indicates that a single radical may initiate several chains through sequential transfer reactions. This mechanism aligns with industrial observations of lower-than-expected molecular weights despite moderate initiator concentrations. Terminal unsaturations introduced by disproportionation are critical for stability, as they can act as reactive sites under thermal or UV stress [125].

Accurate modelling of PVC polymerization must consider conversion-dependent changes in phase composition, chain mobility, and radical diffusion [105,126]. Such models have incorporated chain transfer, backbiting, and termination kinetics using detailed mechanistic frameworks [105]. Advanced computational methods like Gaussian-3 (Møller–Plesset 2)—Radical (G3(MP2)-RAD) and Our-N-layer Integrated Orbital and Molecular mechanics (ONIOM) have also been applied to estimate propagation constants and structural defect pathways, with results in agreement with experimental observations [93,127]. These approaches reveal that defect formation is not random but governed by steric constraints, energetic preferences, and local radical dynamics [125].

Ultimately, the development of structure–property relationships in PVC depends on precise control of chain propagation, transfer, and termination processes. By adjusting processing conditions such as initiator concentration, temperature, and monomer feed rates, polymer microstructure and product performance can be tuned to meet specific application requirements.

### 2.3. Linking Polymerization-Derived Microstructure to Pyrolysis: Radical Chain Kinetics and Product Selectivity

This subsection serves as a transition between polymer synthesis and thermal degradation. This structure to reactivity bridge is developed for saturated chain polyolefins, and it is not extended here to aromatic backbone polymers such as PS or carbonate polymers such as PC. It explains how polymerization defined molecular microstructure (chain architecture) constrains radical formation, scission statistics, and chain-transfer/termination tendencies during pyrolysis, thereby affecting degradation kinetics and product selectivity. This structure—reactivity linkage does not imply mechanistic equivalence between polymerization and pyrolysis; it uses chain architecture as the connecting variable between synthesis and degradation. Commercial waste plastics have heterogeneous processing and service histories. Cooling rate, shear during melt processing, and thermal ageing can change oxidation level, additive state, and the density of weak links, which can shift apparent onset temperature and kinetics. In this review, polymerization derived chain architecture is treated as a baseline descriptor, while processing and ageing history is treated as an uncertainty layer that can widen the observed behavior around that baseline.

In this subsection, noncatalytic thermolysis is treated as the baseline degradation mechanism, while catalytic upgrading over zeolites and additive assisted routes such as Ca based dechlorination are treated as intervention layers that can shift selectivity beyond the baseline. Quantitative generalization is limited because reported yields depend strongly on reactor type, heating program, residence time, pressure, and additives. Solid-state crystallinity is sometimes reported for commercial grades as an accessible proxy correlated with linearity/branching, however, it depends on processing history and is not preserved once the polymer is molten. Coordination polymerization of ethylene with Ziegler–Natta or metallocene catalysts typically produces HDPE with short-chain branching (SCB) in the range of roughly <0.1–2 branches per 1000 carbon atoms and crystallinity around 60–80% [128,129,130]. High-pressure free radical polymerization yields LDPE with about 6–12 SCB/1000 C and a reduced crystallinity of ~30–50% [128]. Stereospecific coordination systems for PP generate isotactic grades with >90–99% meso pentad content and crystallinity of ~50–70%, in contrast to atactic PP with negligible crystallinity [131,132,133]. LLDPE is produced mainly by coordination copolymerization of ethylene with alpha olefin comonomers, which generates controlled short chain branching with minimal long chain branching, so it should be treated as a distinct packaging relevant polyethylene grade rather than merged into LDPE. In PVC, radical polymerization conditions and conversion history determine the fraction of labile structural defects (allylic and tertiary chlorides, head-to-head units, chain-end unsaturations), which may range from below 1% up to a few percent of repeat units and dominate the onset and rate of dehydrochlorination [134,135]. These relationships are summarized in Table 1, which compares polymerization method, branching level, tacticity, and molecular weight distribution for representative commercial PE-LD/LLD and PE-HD/MD, PP, and PVC grades.

The selection of polymerization mechanism, catalyst system and operating conditions quantitatively fixes the accessible microstructural window and therefore constrains the pyrolysis product spectrum. In practice, catalyst identity (Ziegler–Natta vs. metallocene), use of hydrogen, initiator type and dose, reaction temperature and conversion can all be expressed as control variables that tune branching degree, tacticity, crystallinity, molecular weight distribution and defect content [50,130]. Table 2 organizes these relationships for representative coordination and radical systems, explicitly linking polymerization control knobs to microstructural descriptors and to the resulting changes in wax, olefin and dehydrochlorination behaviour. The selectivity shifts summarized in Table 2 are compiled from literature sources that use different reactor configurations and operating windows, and from materials with different additive packages. The table is intended to show directionality of structure and reactivity links under comparable mechanistic regimes, while absolute yields remain sensitive to process severity, residence time, and formulation specific additives or contaminants.

**Table 2:** Polymerization control: detailed quantitative effects on chain architecture and associated trends in pyrolysis selectivity. ↑ indicates an increase in the corresponding parameter relative to the reference condition; ↓ indicates a decrease.

Polymerization Control (Specific Agents/Conditions)	Chain Architecture Descriptor (Molecular)	Pyrolysis Conditions	Pyrolysis Product Selectivity	Rationale	Refs.
Ziegler–Natta catalyst (TiCl_4_/AlR_3_), ↑ H_2_ cofeed (PE)	SCB ↓ (0.1 → 0.02/1000 C); linear sequence fraction ↑ (60 → 75%); Mw ↓ (by up to 50%)	Continuous conical spouted bed reactor: inert nitrogen atmosphere, heating rate 10 °C per min atmospheric pressure, reactor temperature 450 to 600 °C, polymer feed rate about 1 g per min, nitrogen spouting flow about 11 L per min, short vapor residence time	Wax yield +10–15%; olefin yield −10%	H_2_ acts as a chain transfer agent, shortening chains and increasing linearity and branching density; fewer branches and more linear segments favour paraffinic wax formation during pyrolysis.	[54,129,130,142]
Metallocene catalyst (e.g., Cp_2_ZrCl_2_/MAO, PP, PE)	Tacticity ↑ (meso pentad > 99% for PP); Mw/Mn ↓ (6 → 2); defects isolated	Fixed bed reactor: inert nitrogen environment, heating rate 10 °C per min, reactor temperature 500 to 600 °C, isothermal hold 30 min, effective pyrolysis duration about 5 to 10 min depending on temperature.	BTX (catalytic) yield +15–20%; higher wax fractions	Single site control produces highly uniform, stereoregular chains, enhancing the fraction of long, substituted segments that aromatize and survive as heavy waxes in pyrolysis.	[130,143]
Peroxide initiator (e.g., di tert-butyl peroxide, LDPE), ↑ concentration, T ↑	SCB ↑ (6 → 12 /1000 C atoms); branch point density increases, including tertiary carbon sites	Fixed bed reactor: inert nitrogen environment, heating rate 10 °C per min, reactor temperature 500 to 600 °C, isothermal hold 30 min, no carrier gas flow during pyrolysis, effective reaction time about 5 to 10 min.	Light olefin yield +20%; wax yield −15%	Peroxide initiated radical polymerization generates more branching and amorphous content; branched sites undergo β-scission more readily, giving shorter C_2_–C_4_ olefins.	[144,145,146]
PVC via free radical route (e.g., AIBN), ↑ conversion/temperature	Labile defects ↑ (<1 → 5/1000; tertiary/allylic Cl, HTHT units)	Batch tube reactor: isothermal 530 °C, residence time 25 min, inert atmosphere, heating rate 15 °C per min	Dehydrochlorination T ↓ −30 °C; aromatics/char yield +10%	Higher conversion or temperature increases labile Cl—containing defects, lowering the barrier for HCl elimination and promoting backbone crosslinking and aromatization.	[21,134,135,147]

Typical Ziegler–Natta polyethylene systems employ titanium tetrachloride (TiCl_4_) with a cocatalyst, AlR_3_ [54]. Additional hydrogen increases chain-transfer frequency, shortens chains and favours more linear, crystalline structures; such feeds degrade more slowly and accumulate as long-chain paraffinic waxes, while reduced branching suppresses short-olefin formation [129,130]. Metallocene catalysts such as Cp_2_ZrCl_2_ activated with MAO generate highly uniform chains; in PP, this produces very high isotactic indices (meso pentad > 99%), which in catalytic pyrolysis often translate into BTX yields above ~35 wt% over zeolites and into a larger fraction of heavy liquids [68,74].

Peroxide-initiated LDPE, produced under high pressure and temperature, responds strongly to initiator concentration and temperature [144,146]. Free radical PVC synthesis with initiators such as AIBN shows an analogous control lever: higher polymerization temperature or greater conversion increases the population of head-to-head linkages and tertiary or allylic chloride sites, which act as weak points. These defects enable HCl elimination at lower temperatures and leave conjugated backbones that readily crosslink and aromatize, increasing char and aromatic yields in thermal degradation [21,134,135,147]. Together, the relationships in Table 1 and Table 2 provide a quantitative bridge from polymerization operation (choice of catalyst and initiator, use of H_2_, temperature, and conversion) to microstructure, and on to the direction and magnitude of changes in wax, light olefin, BTX, and dehydrochlorination behaviour.

These microstructural parameters translate into measurable differences in pyrolysis product distributions under technologically relevant conditions. At moderate severities typical for plastic waste valorization (e.g., 450–600 °C, inert or vacuum atmosphere, short vapor residence times), linear HDPE grades with low SCB and high crystallinity generally produce a condensable oil/wax fraction on the order of 50–80 wt%, dominated by n-alkanes and α-olefins in the C_10_–C_30_ range, with the remainder as permanent gases [137,138,148]. Under comparable non-catalytic conditions, branched LDPE grades (≈6–12 SCB per 1000 C atoms) usually yield only ~35–45 wt% oils/waxes, while the fraction of light olefins (C_2_–C_4_) increases roughly into the 40–60 wt% range of total products [128,149]. Representative studies show that increasing SCB content from around 2 to around 8 branches per 1000 carbons can reduce wax/oil yields by about 10–15% and increase light olefin yields by roughly 15–20%, demonstrating that branching generated during radical polymerization is a first-order design variable for gas vs. liquid selectivity in polyethylene pyrolysis [148]. Under the same severity and residence time window, LLDPE is expected to show intermediate selectivity, compared with HDPE it shifts toward higher light olefin formation due to higher short chain branching, while compared with LDPE it preserves a larger oil and wax fraction because it lacks the highly branched long chain architecture typical for high pressure LDPE. These quantitative trends are summarized in Table 3, which lists typical ranges of onset temperature, apparent activation energy, and product yields (oil/wax, light olefins, and BTX) for PE HD/MD, PE LD/LLD, isotactic PP, and PVC under comparable pyrolysis conditions, with and without HZSM-5.

**Table 3:** Representative pyrolysis performance ranges for HDPE, LDPE, isotactic PP, and PVC. Onset temperature and $E_{a}$ depend on TGA heating rate. Yield ranges refer to 450 to 600 °C under inert or vacuum atmosphere with short vapor residence times of about 0.5 to 5.6 s.

Polymer	Pyrolysis Onset T (°C)	E_a_ (kJ mol^−1^)	Basis Conditions (Kinetics and Yields)	Oil/Wax Yield 450–600 °C (%)	Olefin Yield (%)	BTX Yield w/o HZSM-5 (%)	BTX Yield w/HSZM-5	Refs.
HDPE	400–440	222–270	TGA under inert gas, heating rate 5 to 30 K min^−1^; reactor pyrolysis at 450 to 600 °C, inert or vacuum atmosphere, vapor residence time 1.4 to 5.6 s, catalyst polymer ratio (mass): 33:1	50–80	30–45	<10	25–35	[137,150,151]
LDPE/LLDPE	410–475	190–240	TGA under inert gas, heating rate 5 to 30 K min^−1^; reactor pyrolysis at 450 to 600 °C, inert or vacuum atmosphere, vapor residence time at or below 0.5 s for liquid rich operation, catalyst polymer ratio (mass): 10:1	35–45	45–60	10–20	30–40	[128,138,149]
PP (isotactic)	370–420	240–301	TGA under inert gas, heating rate 5 to 30 K min^−1;^ reactor pyrolysis at 450 to 600 °C, inert or vacuum atmosphere, vapor residence time 1.4 to 5.6 s, catalyst polymer ratio (mass): 80:1	40–60	35–50	15–20	35–53	[132,152,153]
PVC	270–320 (dehydrochlorination)	131–199	TGA under inert gas, heating rate 5 to 30 K min. Increasing heating rate from 5 to 30 K min^−1^ shifts the main decomposition peak from about 266 °C to 307 °C inert atmosphere. Product yields depend strongly on residence time and secondary reactions under the reactor conditions reported in the cited studies. Catalyst polymer ratio (CaO/Cl): 1:2	10–20	<5	<5	<10	[134,135]

Solid state crystallinity is a morphology descriptor influenced by processing history. It is not used here as a primary mechanistic driver of molten phase pyrolysis. When mentioned elsewhere, it is treated only as a proxy correlated with chain architecture.

From a mechanistic viewpoint, this behavior is consistent with the scission statistics of linear versus branched segments. In molten polyethylene, long linear sequences (typical of HDPE grades with low SCB) tend to generate longer-lived secondary radicals and favor β-scission pathways that yield long-chain fragments and α-olefins, preserving heavier condensables at moderate severity. In contrast, higher SCB/LCB levels (typical of LDPE) increase the frequency of substituted radical sites and intramolecular backbiting, promoting shorter fragments and higher light-olefin yields. Because pyrolysis proceeds after melting, semicrystalline morphology is not treated as a primary mechanistic control variable in this regime. Transport effects are more appropriately discussed via melt viscosity and diffusion, which depend mainly on molecular weight and branching [151,154]. During pyrolysis, Gel Permeation Chromatography (GPC) and Nuclear Magnetic Resonance (NMR) monitoring show that linear segments are cleaved more rapidly than densely branched regions, with a characteristic decrease in maximum segment length from on the order of 100 carbon atoms to below ~50 carbon atoms early in the degradation, while branch-rich regions show higher tendencies for crosslinking and char formation [128]. This difference in local reactivity explains why LDPE produces higher proportions of light olefins and branched hydrocarbons at the expense of heavy waxes under identical thermal histories.

Differences in apparent onset temperatures and activation energies reported across commercial PE/PP grades are often discussed alongside solid-state crystallinity; however, crystallinity depends on both chain architecture and processing history and is not preserved once the polymer is molten [132,133]. Differential scanning calorimetry (DSC) shows that HDPE grades with high crystallinity exhibit sharp melting peaks around 130 °C and higher melting enthalpies, while LDPE shows broader melting ranges (≈70–110 °C) and lower enthalpies, reflecting microstructural heterogeneity [128]. Apparent activation energies and onset temperatures reported by TGA vary across commercial grades primarily because chain architecture and molecular weight distributions differ, including branching density, defect motifs, and dispersity. Under comparable thermal programs, more linear and higher molecular weight feeds tend to preserve longer fragments at moderate severity, while more highly branched architectures more readily generate shorter olefins and lighter products [134]. Thus, polymerization control variables such as catalyst family, hydrogen use, comonomer feed, temperature, and conversion are expressed quantitatively in terms of branching, defect content, and molecular weight distribution, which can then be linked to wax versus gas trends under specified pyrolysis conditions [136,149,155].

For PP, the density and spatial organization of tertiary carbons, fixed during stereoregular coordination polymerization, strongly promote β-scission and aromatization, especially under catalytic conditions [132]. Isotactic PP with high meso content generally displays higher thermal stability in non-catalytic pyrolysis, with mass-loss onset temperatures often reported around 400–420 °C, whereas atactic-rich grades with lower crystallinity may start to decompose around 350–380 °C [132]. Once degradation proceeds beyond the initial random scission phase, both isotactic and atactic PP produce liquids enriched in branched C_5_–C_12_ hydrocarbons and propylene oligomers, but isotactic grades with more extended crystallizable sequences tend to preserve more high-molecular-weight material at a given conversion [136,156]. When PP is processed over Brønsted-acidic zeolites such as HZSM-5 at ~650–750 °C with short vapor residence times, the high abundance of tertiary carbons and methyl-substituted backbone segments leads to elevated BTX yields: typical values reported are on the order of 35–50 wt% BTX in the liquid fraction, compared with roughly 20–30 wt% for HDPE under similar catalytic conditions [157].

Solid-state NMR and fractionation experiments show that isotactic PP sequences can partially isomerize toward more disordered configurations during heating, with a gradual decrease in isotactic pentad content and growth of mixed or syndiotactic sequences [132,133]. This loss of stereoregularity correlates with a decrease in crystallinity and an acceleration of mass loss at later stages of pyrolysis. Samples that start with higher isotacticity and narrower molecular-weight distributions (typical of metallocene catalysts) maintain their crystalline domains longer and show slower conversion to gases at a given temperature, biasing the product slate toward heavier liquids [133]. Thus, polymerization conditions that define isotacticity and molecular-weight dispersity serve as quantitative levers for controlling the distributions of BTX-rich, paraffinic, and gaseous products in PP pyrolysis.

In PVC, the link between polymerization defects and pyrolysis is particularly direct. Labile structural defects, introduced by radical initiation and propagation under non-ideal conditions (e.g., elevated temperature, high conversion, chain-transfer events), lower the activation energy for HCl elimination and shift the dehydrochlorination temperature window [135]. TGA measurements often show a two-stage mass-loss pattern: a first stage between roughly 270–380 °C dominated by HCl evolution and polyene formation, followed by a second stage at higher temperatures, where the conjugated backbone cracks to aromatics, gases and char. Samples with defect contents below about 1% typically begin releasing HCl at ≈300–320 °C, whereas materials with 3–5% labile defects can show onsets closer to 270–290 °C and higher total HCl evolution rates [134]. At a fixed thermal program, more defective PVC grades frequently yield higher fractions of aromatics and char, and slightly lower fractions of condensable aliphatic liquids, because early dehydrochlorination drives extensive backbone conjugation and crosslinking [158]. In practical terms, initiator selection, reaction temperature, chain-transfer control and conversion limits in PVC polymerization can be viewed as upstream parameters for setting the dehydrochlorination profile, HCl release rate and aromatic/char yield in downstream thermal treatment [159,160].

Process variables interact with this microstructural “baseline” but do not erase it at moderate severity. For HDPE and LDPE, increasing heating rate from slow values (e.g., 10–20 °C min^−1^) to swift values (e.g., flash pyrolysis conditions) generally increases the fraction of light olefins and suppresses secondary cracking of volatiles, yet linear HDPE still tends to produce more long-chain waxes than LDPE at the same temperature and residence time [161,162,163]. Similarly, short vapor residence times (≲0.5 s) favor liquid-rich product slates from LDPE at ~500 °C, whereas longer residence times (>1 s) or stronger acidic catalysts drive increased aromatization and gas formation; however, branched LDPE consistently yields more light olefins and aromatics than linear HDPE under matching conditions because its microstructure furnishes more substituted radical and carbocation intermediates [164,165].

Both vapor residence time and heating rate exert profound, quantifiable influences on product distribution, selectivity, and degradation pathways during polymer pyrolysis. Detailed micro-pyrolysis experiments have shown that for ordinary polyolefins such as HDPE, LDPE, PP and PVC, decreasing the vapor residence time from 5.6 s to 1.4 s at a reactor temperature of 600 °C shifts the product distribution significantly towards heavier waxes and liquid hydrocarbons in the C_5_–C_20_ range. In contrast, extending the vapor residence time strongly favors secondary cracking processes, resulting in a markedly higher yield of light gases and aromatic products [165,166]. For instance, under these conditions, gas yields from HDPE can rise from approximately 30 wt% at short residence times to as much as 80 wt% when the residence time is extended and the temperature is increased to 675 °C. Aromatic liquid yields also increase under more severe conditions, reaching values up to 15 wt%. These shifts in product spectrum due to residence time control are consistent with both laboratory and reactor-scale results, further demonstrating that tuning the residence time distribution in fluidized-bed reactors can shift selectivity by 25% or more toward desired products.

Recent studies show that varying the vapor residence time (typically in the range of 1.4–5.6 s) can significantly alter the product distribution, with shorter residence times favoring heavier waxes, while extended residence times lead to more gaseous and aromatic products [165]. This is especially true at higher temperatures (550–675 °C), where longer residence times promote extensive cracking. These findings are in line with reactor scale experiments where manipulating residence time distribution caused shifts of 10–25% in target product yields.

The heating rate is equally decisive in controlling degradation behavior and the evolution of product slats. At low heating rates, around 5 K min^−1^, the onset and maximum of mass loss are observed at lower temperatures, supporting gradual volatilization and enhanced formation of char and solid residue. As the heating rate increases to values such as 30 K min^−1^, the maximum rate of pyrolytic decomposition is shifted to higher temperatures (e.g., PVC’s T_max_ increases from 539 K to 580 K under these conditions), facilitating more intense gas formation, rapid release of small-molecule volatiles (such as HCl from PVC), and substantially greater yields of light olefins and aromatics [167]. For polyolefins, heating rates up to 1000 °C s^−1^, as achieved in flash or micropyrolysis, can lead to near-complete primary conversion within just a few seconds. Fast heating compresses the pyrolysis interval, maximizes light product recovery, and minimizes secondary reactions [165].

Studies, such as those published in [167], demonstrate that increasing the heating rate (from 5 K min^−1^ to 30 K min^−1^ or even up to 1000 °C s^−1^) reduces the pyrolysis time, enhances the production of gaseous and light olefin products, and decreases the overall formation of heavier hydrocarbons and waxes. Such rapid heating is particularly beneficial for maximizing the recovery of lighter components and minimizing secondary cracking [168].

From a modeling perspective, these trends can be captured by kinetic schemes in which rate constants depend explicitly on microstructural descriptors. Random scission models for HDPE can use a single effective scission probability that reproduces the observed chain-length distribution and C_1_–C_5_ gas composition, whereas LDPE models require distinct rate constants for scission at linear segments versus branch-rich segments and sometimes incorporate parallel crosslinking reactions [169]. In PP, kinetic models that weight β-scission and aromatization pathways by tertiary-carbon fraction and degree of substitution reproduce the higher BTX yields observed for isotactic PP relative to polyethylene under catalytic conditions [170]. For PVC, two-step kinetic schemes with defect-controlled dehydrochlorination followed by polyene cracking account for the strong sensitivity of onset temperature and HCl release rate to small changes in defect content [134].

Polymerization conditions set the microstructure which biases wax oil, light olefin, gas and catalytic BTX tendencies under stated operating windows, while absolute yields remain sensitive to reactor severity, residence time and formulation. In practice, linear HDPE is wax-oriented; branched LDPE favors light olefins; stereoregular PP is BTX-oriented over acidic catalysts; and PVC with engineered defect content demands staged dehydrochlorination with basic sorbents.

Therefore, chain architecture provides a baseline framework for interpreting selectivity trends, while processing history and ageing can widen observed product distributions and shift apparent onset temperature even under similar operating conditions.

Commercial waste plastics are formulated materials and may contain antioxidants and stabilizers, fillers and pigments, plasticizers in PVC, residual catalyst species, and external contaminants. These components can scavenge radicals or promote radical generation, modify acid or redox microenvironments, and change the apparent onset and product distribution under the same nominal reactor temperature. Therefore, the chain architecture discussion in this subsection is treated as a mechanistic baseline, while additive and impurity effects are treated as an overlay that can shift the magnitude and sometimes the direction of selectivity trends

## 3. Mechanistic Pathways and Kinetic Characteristics of Thermal Degradation in HDPE, LDPE, PP and PVC

The following mechanistic descriptions treat chain architecture as the primary substrate level control, while recognising that additives, catalyst residues, and contaminants in waste derived feeds can overlay these pathways by shifting radical chemistry and apparent kinetics.

### 3.1. HDPE

The initiation of thermal degradation in HDPE proceeds via homolytic cleavage of carbon–carbon (C–C) bonds, triggered when the system temperature exceeds the bond dissociation energy threshold. This process results in the formation of primary alkyl radicals that mark the onset of degradation, initiating radical chain reactions across the polymer backbone [80,81,82].

**Figure 6:**
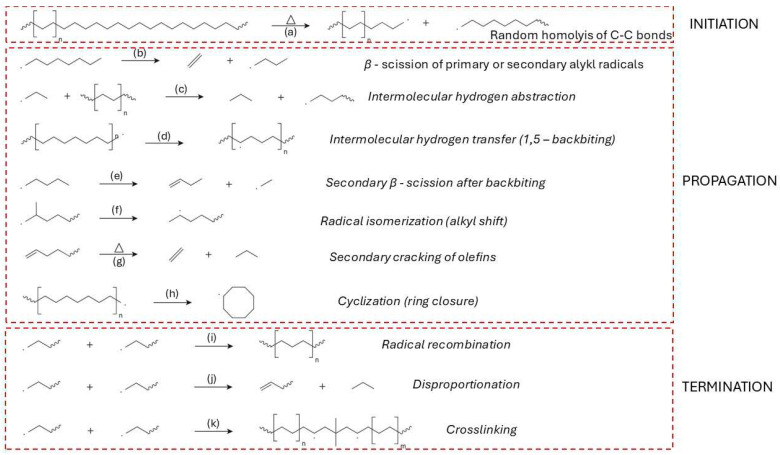
Thermal degradation mechanism of HDPE: (**a**) initiation by random C–C bond homolysis; (**b**) β-scission of primary/secondary alkyl radicals; (**c**) intermolecular hydrogen abstraction; (**d**) intramolecular hydrogen transfer (1,5-backbiting); (**e**) secondary β-scission following backbiting; (**f**) radical isomerization via alkyl shift; (**g**) secondary cracking of olefins; (**h**) cyclization into cyclic radicals; (**i**) radical recombination forming saturated chains; (**j**) disproportionation yielding unsaturated end groups; (**k**) crosslinking via radical coupling between chains.

Early degradation events are predominantly described by a random scission model, wherein each C–C bond along the linear polymer chain has an equal probability of breaking under thermal stress (Figure 6a). This statistical approach successfully captures observed kinetic behavior and molecular weight distributions during pyrolysis, particularly in the absence of branching or catalytic residues [80,82,83]. However, site-specific initiation mechanisms have also been proposed, emphasizing preferential scission at structurally weaker sites—such as allylic positions or residual catalyst centres—where degradation may commence at lower temperatures [84].

Homolytic scission events generate highly reactive radicals that participate in subsequent reactions, such as hydrogen abstraction (Figure 6c), vinyl formation, and β-scission (Figure 6b). These radicals create vinyl and vinylidene end groups, with vinyl groups forming at rates significantly higher than the evolution of ethylene in early degradation stages, suggesting that functional group generation dominates over monomer recovery in the initiation phase [86].

Experimental studies have shown that the initiation step is highly sensitive to molecular weight, crystallinity, and thermal history. In waste derived feeds, prior melt processing and thermal ageing can introduce oxidized sites and additional weak links, which can shift the apparent onset temperature and kinetics. Lower molecular weight fractions degrade more readily due to enhanced chain mobility and reduced thermal stability, while crystalline regions offer greater resistance to bond rupture [81,86,171]. The onset temperature for random scission typically ranges between 400 and 440 °C, although the presence of impurities, chain defects, or catalytic residues can lower this threshold by as much as 100 °C [151].

Kinetic modelling of the initiation phase supports a homolytic mechanism with reported activation energies for HDPE degradation ranging from approximately 222 kJ mol^−1^ to 270 kJ mol^−1^, depending on the method used (e.g., Kissinger or Ozawa-Flynn-Wall and Vyazovkin isoconversional method) and the polymer architecture [151]. These values reflect the energy requirement for C–C bond cleavage and provide a quantitative foundation for process optimization in thermal recycling.

Although the random scission model remains widely accepted, recent work has highlighted the importance of distinguishing between uniformly random (chain reaction) and structurally biased (attack on β-positioned carbon) initiation. These distinctions are particularly relevant for refining pyrolysis models and improving the accuracy of product yield predictions in both open and closed systems [7,81].

Ultimately, the initiation of HDPE thermal degradation is best described as a complex interplay between random homolytic bond scission and site-specific activation, both of which are governed by temperature gradient rate and final temperature. Understanding this initiation step is essential for controlling downstream reaction pathways and optimizing the pyrolytic conversion of HDPE into valuable hydrocarbon fractions.

The propagation phase of HDPE thermal degradation is governed by a complex network of free radical-driven reactions that proceed following the initial homolytic cleavage of C–C bonds. At the beginning (at lower temperatures), the primary mechanism is random scission; as temperatures rise and the concentration of β-carbon positions increases, β-scission becomes the dominant mechanism (Figure 6b). Hypothesis can be supported by the formation of alkene products at higher temperatures of pyrolysis [172].Intermolecular hydrogen abstraction (Figure 6c) is another key step, where radicals extract hydrogen atoms from neighbouring polymer chains or smaller hydrocarbon molecules, thereby generating new radical sites and further driving the degradation process [86,172]. Intramolecular hydrogen transfer (Figure 6d), commonly referred to as backbiting, plays a critical role in creating secondary radical sites that undergo secondary β-scission (Figure 6e), contributing to the diversity of volatile products [171,173].

Radical isomerization, including alkyl shifts, promotes the rearrangement of radical intermediates to more stable configurations (Figure 6f), thereby altering the final product distribution [172,174]. Secondary cracking of olefins, especially under elevated temperatures and prolonged vapor residence times, further reduces long-chain olefins into lighter hydrocarbons (Figure 6g) [151,175].

Cyclization reactions can occur during degradation, where radicals undergo ring closure to form cyclic hydrocarbons (Figure 6h). These cyclic intermediates can subsequently undergo dehydrogenation and further rearrangements, eventually leading to the formation of aromatics and polycyclic aromatic hydrocarbons (PAHs), especially at temperatures above 600 °C [151,172,174,175].

Vinylidene formation is another important reaction pathway, leading to the development of vinyl-type structures through radical-induced elimination reactions, contributing to the unsaturation of the degradation products [151,172]. Minor radical β-scission and recombination equilibria also play a role in controlling the balance between chain fragmentation and radical stability, particularly at elevated temperatures (Figure 6i) [172,176].

Radical-induced disproportionation further contributes to the diversity of degradation products (Figure 6j) by forming stable radicals and smaller hydrocarbons [110]. Additionally, aromatization and PAH formation pathways are significant under severe pyrolytic conditions, as cyclic radicals undergo dehydrogenation and ring rearrangement to form stable aromatic structures [151].

Ring-opening reactions of cyclic hydrocarbons contribute to the formation of linear chain products, further enhancing the diversity of volatile products [151,171]. Dehydrogenation processes, especially at elevated temperatures, increase the unsaturation level in the resulting hydrocarbons and promote the formation of aromatic compounds [171,175,177].

Experimental studies confirm that the progression of propagation reactions and the final product distribution are highly dependent on the thermal environment and residence time. Low temperatures and short vapor residence times favour a broader spectrum of alkanes, alkenes, and waxes, while higher temperatures and longer residence times predominantly yield light hydrocarbons, monoaromatics, and PAHs [172,174].

Oxidative pathways, although not the focus of the propagation phase under inert pyrolysis conditions, may become significant in processing environments such as extrusion, where oxygen exposure leads to the formation of oxidized volatiles, such as aldehydes and carboxylic acids [178]. Additives, including flame retardants and catalytic residues, can also modulate the degradation pathway by accelerating or redirecting radical reactions toward desired product distributions or char formation [179,180].

Overall, the propagation phase in HDPE thermal degradation involves a dynamic interplay of β-scission, hydrogen abstraction, backbiting, isomerization, secondary cracking, cyclization, vinylidene formation, and dehydrogenation reactions. These mechanisms collectively dictate the evolution of product species and govern the molecular weight reduction, unsaturation levels, and aromatic content in the final pyrolysis products.

For HDPE, shown in Figure 6i–k, the termination processes outlined in Figure 5 are strongly influenced by crystallinity and branching [181,182]. Under vacuum and in the absence of oxygen, recombination of macroradicals is favored due to high melt viscosity, contributing to the retention of molecular weight and wax formation [169]. Disproportionation is supported by the formation of vinyl and vinylidene groups observed in product analysis, particularly in branched or low-mobility regions [181,183]. HDPE’s limited unsaturation restricts crosslinking, but the process can be promoted by residual catalyst fragments or increased branching [184]. The balance of these mechanisms dictates the yield of long-chain paraffins, olefins, and the potential for network formation under varying thermal conditions [185].

### 3.2. LDPE

**Figure 7:**
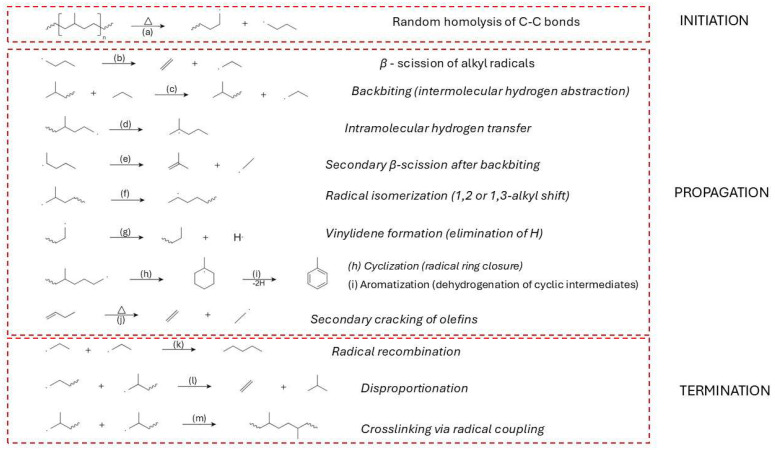
Thermal degradation mechanism of LDPE: (**a**) random C–C bond scission (homolysis); (**b**) β-scission of alkyl radicals; (**c**) backbiting (intermolecular hydrogen abstraction); (**d**) intramolecular hydrogen transfer; (**e**) secondary β-scission after backbiting; (**f**) radical isomerization (1,2 or 1,3-alkyl shift); (**g**) vinylidene formation (H elimination); (**h**) cyclization (ring closure); (**i**) aromatization (dehydrogenation of cyclic intermediates); (**j**) secondary cracking of olefins; (**k**) radical recombination; (**l**) disproportionation; (**m**) crosslinking via radical coupling.

The thermal degradation of LDPE under vacuum initiates through random homolytic scission of C–C bonds along the polymer backbone (Figure 7a). This unimolecular process occurs without oxidative interference and becomes significant at elevated temperatures between 410 and 475 °C, where thermal energy exceeds the bond dissociation threshold of saturated carbon chains [186]. The alkyl radicals generated in this initiation step serve as the starting point for subsequent β-scission and hydrogen abstraction reactions.

Although often modelled as purely random, the cleavage of C–C bonds in LDPE is not uniformly statistical. Thermochemical studies and experimental analyses suggest a preference for scission at structurally weaker sites, such as allylic positions or tertiary carbons, due to their lower dissociation energies [7,81,187]. These preferential sites enable radical formation at lower activation energies and contribute to the early evolution of unsaturated species such as vinyl and vinylidene groups [188].

Upon C–C bond cleavage, primary and secondary alkyl radicals are formed. The stability of these radicals influences their reactivity, with secondary radicals generally being more thermodynamically favored and more likely to propagate the degradation process [186]. The radicals can also undergo stabilization through resonance delocalization or hydrogen abstraction from neighboring chains, forming longer-lived radical intermediates capable of driving further decomposition [189].

The initiation phase also leads to significant structural changes within the polymer matrix. These include the formation of double bonds and unsaturated end-groups, an increase in reactive chain ends, and the production of low molecular weight aliphatic fragments such as alkanes and alkenes [190]. These effects are consistent with both experimental measurements and kinetic simulations, which have reported early-stage molecular weight reduction and volatile product evolution as primary indicators of radical initiation [191].

Additionally, molecular dynamics and variational transition state theory have shown that bond dissociation rates increase with chain length up to decane, after which the effect plateaus. This chain length dependence influences the overall fragmentation rate of the polymer backbone [187]. The propagation phase is mainly driven by β-scission of alkyl radicals, which break adjacent C–C bonds to produce unsaturated chain ends and smaller hydrocarbon fragments [192,193]. After the initial formation of radicals, backbiting through intermolecular hydrogen abstraction (Figure 7c) often occurs, especially in LDPE, because its branched structure allows for closer proximity between radical sites and hydrogen atoms within the same polymer chain [194,195]. Intramolecular hydrogen transfer (Figure 7d) then repositions the radical along the polymer backbone, preparing it for secondary β-scission (Figure 6e), which further breaks down the chain [193,196].

Radical isomerization, through 1,2- or 1,3-alkyl shifts (Figure 7f), enables migration of radical centres to thermodynamically more stable positions, affecting both chain stability and subsequent degradation steps [197]. Vinylidene groups form via the elimination of a hydrogen atom from terminal radicals (Figure 6g), introducing unsaturation into the polymer. Cyclization (Figure 7h), especially in flexible chain segments, results in the formation of cyclic intermediates, which at elevated temperatures undergo aromatization via dehydrogenation (Figure 7i), yielding aromatic compounds such as benzene or substituted aromatics [193].

The olefins generated in the primary degradation steps may undergo secondary cracking (Figure 7j), yielding smaller hydrocarbons such as ethylene, propylene, and butenes, especially at prolonged residence times and elevated temperatures [86,198].

Several additional processes are intertwined with the radical propagation phase. Random chain scission persists throughout degradation, generating fragments of varying molecular weights [198,199]. Depolymerization, characterized by the stepwise loss of monomer units, further reduces the polymer chain length and contributes to the formation of low-molecular-weight products [200]. Under specific thermal conditions, crosslinking between polymer chains may also occur (Figure 7m) [100].

It has been demonstrated that the degradation rate transitions from zero-order to first-order kinetics as the temperature increases from ~340 °C to above 425 °C, indicating the activation of radical-driven propagation reactions above a critical temperature threshold [192]. The relevance of these radical propagation pathways extends to practical applications such as pyrolytic recycling and the thermal stability of polyethylene-based materials. The formation of cyclic intermediates, aromatics, and PAHs, although more prevalent under severe pyrolytic conditions, can have significant environmental and material performance implications [193,196,198].

In summary, the propagation phase of LDPE thermal degradation under vacuum is defined by a series of interconnected radical processes, including β-scission (Figure 7b,e), hydrogen abstraction (Figure 7c,d), radical isomerization (Figure 7f), vinylidene formation (Figure 7g), cyclization (Figure 7h) and aromatization (Figure 7i).

Termination in the thermal degradation of LDPE under vacuum proceeds through multiple radical-driven pathways. Radical recombination (Figure 7k) is a key termination pathway that leads to saturated covalent bonds and the loss of radical activity. Disproportionation (Figure 7l) results in one saturated and one unsaturated chain by hydrogen transfer between radicals [200,201,202]. Crosslinking (Figure 7m) occurs at high temperatures in the melt phase, forming a three-dimensional network, but to a minor extent [100,203,204].

### 3.3. PP

**Figure 8:**
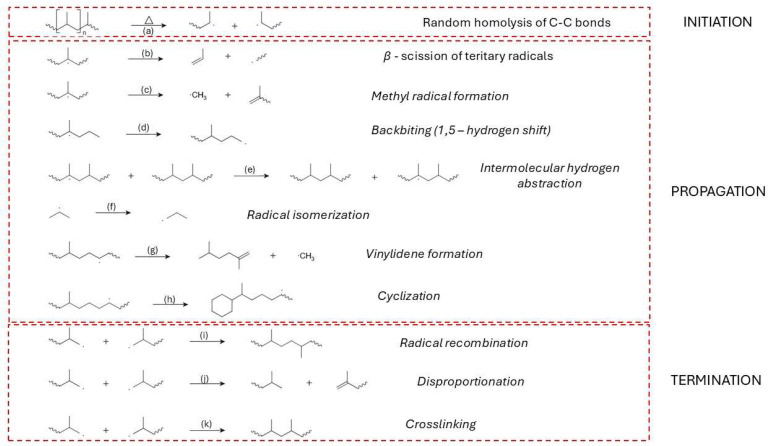
Reaction mechanism of the thermal degradation of PP. (**a**) Random homolysis of C–C bonds; (**b**) β-scission of tertiary radicals; (**c**) methyl radical formation; (**d**) backbiting (1,5-hydrogen shift); (**e**) intermolecular hydrogen abstraction; (**f**) radical isomerization (1,2/1,3-alkyl shifts); (**g**) vinylidene formation (terminal hydrogen elimination); (**h**) cyclization via radical-induced ring closure; (**i**) radical recombination forming saturated chains; (**j**) disproportionation yielding one saturated and one unsaturated chain; (**k**) crosslinking through radical coupling between polymer chains.

The initiation phase of PP thermal degradation under vacuum is governed predominantly by random homolytic cleavage of carbon–carbon (C–C) bonds along the polymer backbone (Figure 8a). This unimolecular process leads to the generation of primary, secondary, and particularly tertiary alkyl radicals, which serve as reactive intermediates for subsequent propagation pathways [205,206,207].

Homolytic scission in PP occurs statistically along the polymer chain. Still, structural factors such as the presence of methyl substituents adjacent to tertiary carbons in isotactic PP significantly influence the selectivity and stability of the resulting radicals. The formation of tertiary-centred radicals is favored both thermodynamically and kinetically due to the stabilization conferred by the polymer’s tacticity and branching pattern [208,209,210].

Computational and experimental analyses confirm that although scission is initially random, the resulting product distribution and radical reactivity are highly non-random. Product analysis from PP pyrolysis shows a high prevalence of branched olefins—e.g., 2,4-dimethyl-1-pentene—which points to a mechanistic preference for radical transfer to tertiary centres and subsequent β-scission at these sites (Figure 8b) [6,208]. This implies that radical transfer, both intra- and intermolecular, plays a central role during initiation, allowing chain mobility and localized reactivity to guide the degradation pathway.

The activation energy for initiation via random backbone cleavage is reported to be approximately 301.3 kJ mol^−1^, while secondary side-scission processes (e.g., at tertiary radicals) may proceed with significantly lower barriers (~123.9 kJ mol^−1^) (Figure 8b) [206]. These energy differences support a dual-mechanism model: initial radical formation requires substantial thermal input, while subsequent reactions proceed via more accessible routes. This aligns with PHASE-based kinetic studies showing a sharp two-stage reactivity pattern, where a slower transformation of unsaturated residues and char precursors follows an initial rapid degradation phase [211].

Mechanistic modelling of PP pyrolysis, incorporating the majority reactions and species, validates the dominance of random C–C homolysis in initiating degradation, especially at elevated temperatures above 380 °C (Figure 8a) [207,208,212]. The transition from weak-link activation at lower temperatures (200–350 °C) to homolysis-dominated initiation at higher temperatures (>380 °C) is well supported by changes in volatile product yields and degradation kinetics [12,213].

Additionally, initiation events are closely linked to hydrogen-abstraction processes, which either stabilize radicals or generate new radical species (Figure 8e). These transfer reactions are facilitated by the presence of labile tertiary hydrogen atoms, which are abundant in isotactic PP, enhancing the likelihood of radical rearrangement and unsaturation formation (Figure 8d) [6,12,209].

Environmental conditions, particularly vacuum and inert atmospheres, affect the onset and rate of initiation. Under vacuum, the absence of oxygen inhibits oxidative side reactions, thereby amplifying the contribution of homolytic scission and suppressing low-temperature degradation pathways [206,212,213].

Spectroscopic and molecular weight analyses further confirm the initiation process. A measurable decrease in intrinsic viscosity correlates with chain scission extent, and FTIR observations reveal increasing carbonyl and vinyl group concentrations as degradation progresses [210]. These findings indicate that unsaturation and chain-end functionalities emerge early during pyrolysis, reflecting the impact of radical generation and migration during the initiation stage.

The propagation phase is driven by a complex series of radical-mediated reactions that evolve from the primary macroradicals formed during initiation. Once generated, these radicals—primarily tertiary in structure due to the methyl-substituted backbone of PP—undergo β-scission, a dominant propagation step that cleaves C–C bonds adjacent to the radical centre (Figure 8b). This process results in the formation of shorter hydrocarbon fragments and new secondary radicals, effectively sustaining the degradation cascade. The β-scission of tertiary radicals is thermodynamically favorable and leads to the formation of unsaturated hydrocarbons such as propylene, 2-methyl-1-pentene, and other branched olefins, which are frequently detected among the pyrolysis products [12,156,207,208,214,215].

Methyl radicals are also formed during this process, either through side-group fragmentation or as secondary products of β-scission events (Figure 8c). These small radicals are highly reactive and play a key role in product diversification and hydrogen abstraction reactions. The system further propagates via backbiting reactions, particularly the 1,5-hydrogen shift, in which a hydrogen atom is transferred intramolecularly to a radical site via a six-membered transition state (Figure 8d). This leads to the formation of more stable radicals, often tertiary, which are prone to subsequent β-scission. The backbone structure of PP facilitates backbiting and is especially active near chain ends or in regions with local chain mobility [208,215].

In parallel, intermolecular hydrogen abstraction allows radicals to abstract hydrogen atoms from neighboring polymer chains (Figure 8e). The hydrogen abstraction step generates new radical centres while stabilizing the abstracting radical, thereby spreading radical activity throughout the polymer matrix. This mechanism contributes significantly to the degradation process, particularly under high-radical-density conditions typical in vacuum pyrolysis [6,12,209,215]. Alongside hydrogen abstraction, radical isomerization also plays a substantial role in redistributing radical centres (Figure 8f). This occurs through hydrogen shifts, such as 1,2- or 1,3-transfers, enabling the radical to migrate to more thermodynamically stable sites, usually adjacent to tertiary carbon atoms. The resulting rearrangements increase the variety and stability of degradation intermediates and influence the composition of the resulting product pool [207,216].

Terminal double bonds are indicative of end-chain scission and are commonly observed among PP pyrolysis products, especially in the form of monoolefins (Figure 8g) [208]. The formation of allylic radicals, stabilized through resonance with adjacent double bonds, is another critical aspect of propagation. These radicals contribute to the formation of conjugated dienes and persist longer in the radical pool, influencing both the rate and the extent of subsequent decomposition steps [207,217].

Cyclization reactions, although less prevalent, are recognized in PP degradation, particularly under thermal conditions that permit chain folding and intramolecular radical attack (Figure 8h). The formation of cyclic intermediates or products has been supported by the frequent overrepresentation of specific hydrocarbon fragments such as C_9_ and C_15_, which align with mechanistically favored ring closure and β-scission events involving cyclic transition states [207]. In some cases, these intermediates can further undergo dehydrogenation to yield aromatic products, although such transformations are secondary in PP due to the saturated nature of its backbone.

The accumulation and interaction of radicals during propagation also enable recombination reactions, particularly at elevated concentrations or at later stages of degradation (Figure 8i). Radical recombination leads to the formation of heavier, more complex hydrocarbons and can serve as a partial termination route; however, in a vacuum, the low pressure favors continued radical propagation over termination. The product distribution observed in PP pyrolysis under vacuum—especially the dominance of C_5_–C_15_ olefins—reflects the combined influence of β-scission, backbiting, hydrogen abstraction, isomerization, and occasional cyclization steps [12,214,218].

Overall, the propagation phase of PP thermal degradation under vacuum is governed by an interdependent network of radical reactions. These reactions are modulated by chain structure, stereochemistry, and the thermal environment, and ultimately determine the rate of molecular weight reduction and the identities of volatile degradation products. The predominance of specific product series (e.g., hydrocarbons with carbon numbers divisible by three) confirms that propagation is not entirely random but influenced by preferred radical transfer routes and chain segment stabilities. The combination of detailed mechanistic modelling and empirical product analysis provides robust evidence that radical propagation in PP involves multiple pathways [171].

In PP pyrolysis (Figure 8i–k), the exact fundamental termination mechanisms apply as shown in Figure 5. Still, the prevalence and product profile differ due to PP’s tertiary carbon centers and methyl side groups. Recombination is encouraged at elevated temperatures and radical concentrations, boosting alkane and alkene yields [12,94,214]. Disproportionation contributes to vinylidene and terminal alkene formation, increasing with radical mobility [219,220]. Crosslinking is less frequent in PP than in HDPE, yet it can become significant when radical density is high or under energy input, such as radiation, affecting thermal stability and rigidity [12,208]. These structural distinctions and the environmental context thus shape product distribution in PP [6,86,205].

Altogether, these termination mechanisms act in tandem to regulate the balance between continued radical propagation and product stabilization. Radical recombination is the most direct path to neutralize radicals and dominates at high radical concentrations. Disproportionation introduces unsaturation into the degradation products and promotes olefin formation, while crosslinking alters the polymer’s physical network, influencing its thermal and mechanical properties. The relative prevalence of each pathway is dictated by temperature, radical concentration, and molecular mobility—all of which are modulated under vacuum conditions typical of PP pyrolysis.

### 3.4. PVC

Under vacuum, the thermolysis of PVC is governed by the polarity and relative weakness of the C–Cl bond and by the heterogeneous distribution of labile structural conditions that seed chemical change. Cleavage events originate preferentially at allylic and tertiary chlorines, head-to-head linkages, and other polymerization defects where electronic (resonance, hyperconjugation) and steric factors reduce the barrier for C–Cl scission [221,222,223]. A syn-periplanar >CH–CCl< geometry aligns σ_C-H_ and σ_C-Cl_ orbitals to support concerted 1,2-elimination through a four-center transition state; in tertiary environments, six-center concerted pathways are frequently accessible and display substantially lower activation energies (≈173.6 kJ mol^−1^) than regular repeat units (≈230.5 kJ mol^−1^) [224,225]. Quantum–chemical calculations, Modified Neglect of Diatomic Overlap/Austin Model 1/Density Functional Theory (MNDO/AM1/DFT), and model compound studies reproduce these energetic trends and rationalize why carefully purified (reprecipitated) PVC shows more uniform kinetics: the average length of initially dehydrochlorinated segments (l_avl_) typically spans ~4–12, tracking the density and topology of labile sites [226]. Microstructure (tacticity, branching) modulates the population and spatial disposition of tertiary chlorines; temperature and Lewis-acid coordination to chlorine further polarize and weaken C–Cl bonds, advancing the onset and rate [223,226,227]. Because evolved HCl is efficiently removed under vacuum, bulk acid does not accumulate, autocatalysis is damped, and molecular (ionic) elimination dominates [221,228].

**Figure 9:**
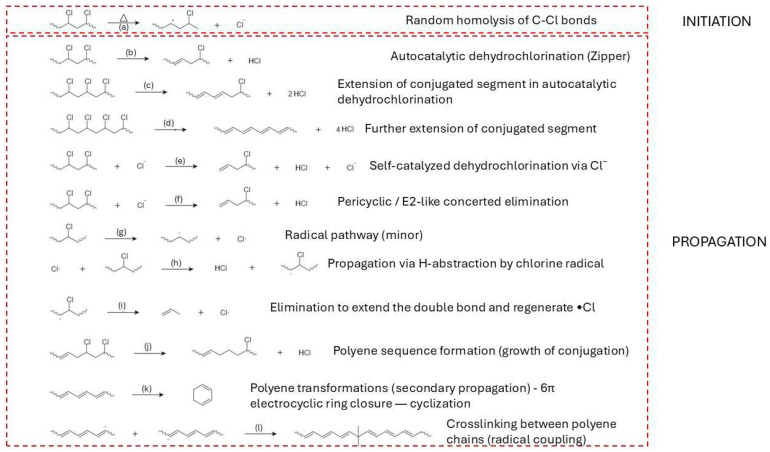
Initiation and propagation steps in the thermal degradation of PVC. The sequence includes (**a**) random homolysis of C–Cl bonds, which generates initiation sites, (**b**) autocatalytic dehydrochlorination via the zipper mechanism, (**c**) extension of the conjugated segment in autocatalytic dehydrochlorination, (**d**) further extension of conjugation along the chain, (**e**) self-catalyzed dehydrochlorination via chloride ions, (**f**) pericyclic/E2-like concerted elimination, (**g**) the minor radical pathway, (**h**) propagation through hydrogen abstraction by chlorine radicals, (**i**) elimination steps extending the double bond with regeneration of Cl radicals, (**j**) polyene sequence formation with growth of conjugated domains, (**k**) polyene transformations through secondary propagation including 6π electrocyclic ring closure (cyclization), and (**l**) crosslinking between polyene chains via radical coupling.

The mechanistic map captured in Figure 9a–l begins with Figure 9a, which shows random homolysis of C–Cl bonds as a minor but mechanistically relevant entry that creates macroradicals on the backbone together with chlorine radicals (Cl^•^) [96,97]. The dominant pathway, however, is the autocatalytic zipper dehydrochlorination illustrated in Figure 9b–d: successive eliminations of HCl from adjacent –CH_2_–CHCl– pairs progressively extend a conjugated polyene segment along the same chain—growth in conjugation length, not in molecular weight [224,227,228,229]. Two intrinsic, non-radical channels operate alongside the zipper: Figure 9e depicts self-catalyzed dehydrochlorination via chloride (Cl^−^), an E2-like process in which Cl^−^ is regenerated, and Figure 9f shows a pericyclic/E2-like concerted elimination that proceeds through a six-center transition state when stereogeometry permits [13,15,230,231]. Although ionic routes dominate under vacuum, Figure 9g–i summarizes a minor radical loop: homolytic C–Cl rupture forms macroradicals and Cl^•^ (Figure 9g), Cl^•^ abstracts hydrogen from saturated segments to produce HCl and new macroradicals (Figure 9h), and β-elimination on those radicals extends the double bond while regenerating Cl^•^ (Figure 9i) [226,228,232]. As the zipper advances, Figure 9j emphasizes the buildup of polyene sequences (–CH=CH–)*_n_*, which act as platforms for secondary propagation processes. Most notably, Figure 9k shows a thermally allowed 6π-electrocyclic ring closure of a triene fragment to cyclohexadiene-like motifs (thermal disrotatory pathway consistent with Woodward–Hoffmann rules), thereby embedding cyclic/aromatic precursors into the backbone [221,230,233]. Figure 9l then highlights interchain radical coupling between polyene segments (crosslinking), which—together with concurrent cis–trans isomerization that biases the π-array toward lower-energy trans configurations—progressively immobilizes and stiffens the material [233,234,235].

Kinetic observations align with this picture. Early conversion is well described by apparent first-order behavior with respect to the concentration of labile motifs, consistent with defect-initiated progress of the zipper [160,221,230]. β-Chloroallyl and polyenyl-chloride sequences are kinetically privileged, accelerating subsequent HCl loss; where HCl removal is locally imperfect, the acid can lower the enthalpy of dehydrochlorination by up to ~110 kJ mol^−1^ in susceptible fragments, transiently increasing the rate [221,228,232,236]. Polyenyl segments generally eliminate faster than β-chloroallyl motifs, establishing a moving hierarchy of reactivity as conjugation grows [232]. Morphology (crystallinity, plasticizer content) modulates segmental mobility and the diffusion of HCl and Cl^−^, thereby shaping l_avl_ and the spatial pattern of zipper advance [13,160,221,226]. There is also evidence that cation-radical species formed by interactions of polyenes with HCl can abstract hydrogen and sensitize unreacted sequences, creating localized autocatalytic pockets even when radical concentrations are globally low [160].

**Figure 10:**
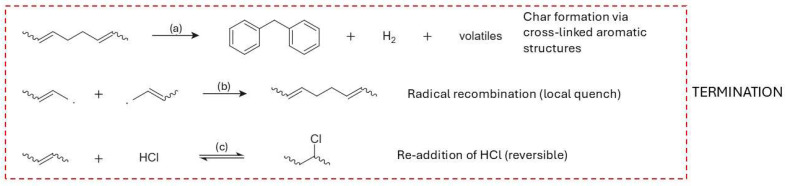
Termination steps in the thermal degradation of PVC. The processes comprise (**a**) char formation through the development of cross-linked aromatic structures, (**b**) radical recombination leading to local quenching of chain reactivity, and (**c**) reversible re-addition of HCl to unsaturated sites. These termination pathways highlight how conjugated polyenes evolve toward stable aromatic char residues, with small fractions undergoing reversible reactions or quenching events.

As conjugated domains mature, the chemistry tilts toward stabilization pathways summarized in Figure 10a–c. Figure 10a depicts char formation: long polyenes cyclize (via repeated 6π closures), dehydrogenate, and crosslink to yield condensed aromatic frameworks that are carbon-rich, thermally robust, and poorly volatile [12,221,233]. Transition metal additives such as NiO or MoO_3_ can reinforce this evolution by catalyzing intermolecular chlorine loss and producing NiCl_2_ in situ, which promotes further unsaturation and raises crosslink density, thereby increasing char yield while suppressing volatiles [237,238]. Figure 10b shows radical recombination—bimolecular coupling of macroradicals formed during propagation—to generate new C–C bonds that locally quench reactivity and add crosslinks to the network [221,233]. Figure 10c illustrates reversible HCl-addition to double bonds; under efficient vacuum, this route is minor, but where HCl is not entirely removed, it can transiently regenerate –CH–CH(Cl)– units and interrupt further eliminations, thus moderating the pace of unsaturation [221,233,236]. At elevated temperatures, the late-stage polyene backbone can also fragment, releasing light alkenes and aromatic species; radical cyclization/dehydrogenation of these aromatics leads to PAHs, a fraction of which volatilizes while the remainder integrates into the growing char network [239,240].

Taken together, Figure 9 and Figure 10 outline a coherent mechanistic trajectory under vacuum: selective C–Cl scission at structurally weak sites seeds unsaturation; conjugation grows chiefly by zipper dehydrochlorination, aided by self-catalyzed and concerted E2-like eliminations and accompanied by minor radical cycles (Cl^•^, macroradicals); polyenes then reorganize via 6π electrocyclizations, cis–trans isomerization, and interchain radical coupling; and termination proceeds by aromatization and crosslinking into carbon-rich char, with local radical recombination and small, reversible HCl additions shaping fine details of the residue. This mechanistic synthesis not only rationalizes kinetic signatures (first-order on labile motifs, sensitivity to l_avl_, morphology, and HCl removal) but also provides levers for stabilization strategies in processing—namely, controlling defect populations and microstructure, enhancing acid scavenging and HCl evacuation, and judiciously using Lewis-acid inhibitors or transition metal traps to steer the balance between volatiles and char [221,233,241].

## 4. Tuning Thermal Degradation Pathways with Potential Catalysts and Initiators: Lowering Onset and Enabling Selective Termination

### 4.1. PE, PP and PVC: Influence of Catalysts, Initiators, and Termination Procedures

#### 4.1.1. Catalyst Effect for HDPE, LDPE, PP and PVC Pyrolysis

Catalytic pyrolysis represents a critical pathway for upgrading plastic waste streams, particularly HDPE, LDPE, PP, and PVC. Within the polyethylene family discussed in this section, MDPE is treated together with HDPE as the PE HD/MD group, and LLDPE is treated together with LDPE as the PE LD/LLD group. The introduction of tailored catalysts lowers degradation onset temperatures, accelerates reaction kinetics, and enables control of product selectivity, whereas in PVC catalytic strategies additionally focus on dehydrochlorination, polyene stabilization, and HCl management. The following synthesis integrates current literature across these four major polymers, highlighting mechanistic pathways, catalyst classes, and practical challenges.

In the case of HDPE, catalytic pyrolysis consistently lowers decomposition temperatures and improves reaction rates compared to pure thermal cracking. Zeolites dominate catalytic performance, with HZSM-5 and Hydrogen-form Y-type Zeolite (HY) being the most studied. HZSM-5, with its strong Brønsted acidity and shape-selective micropores, enriches light olefin yields such as propylene and butenes, whereas HY, due to its larger pore size, enhances aromatic selectivity [242,243,244]. Adjustment of the SiO_2_/Al_2_O_3_ ratio in HZSM-5 strengthens acidity and has been reported to boost olefin production up to 58 wt% [243]. Mesoporous materials such as Mobil Composition of Matter 41 (MCM-41), Santa Barbara Amorphous-15 (SBA-15), and hierarchical Beta expand diffusion channels, and when acidified, shift product distributions toward branched and aromatic hydrocarbons [245]. Metal oxides, including MgO, ZnO, and Mg_x_AlO_γ_, are effective in yielding paraffin-rich liquids with superior catalyst stability, with Mg_x_AlO_γ_ particularly suited to mixed-plastic feedstocks [246,247]. Natural clays such as bentonite and kaolin are less active but remain economically favorable alternatives [248]. Transition metal modifications, exemplified by Ni/Y, lower activation energies and extend catalyst life, though often with reduced initial activity [242,249]. Across catalysts, kinetic studies confirm significant activation energy reductions, enabling accelerated cracking at lower temperatures [250,251]. Catalyst choice steers distribution sharply: HZSM-5 promotes light olefins, Hydrogen-form Ultra-Stable Y-type Zeolite (HUSY) and Hydrogen-form Beta Zeolite (HBEA) increase aromatics and isoparaffins, while thermal pyrolysis favors 1-olefins and n-paraffins [11,244,252,253]. Two-step configurations, where thermal pyrolysis is followed by catalytic upgrading, further enhance selectivity and minimize coking [243]. Persistent challenges include coke deposition and rapid deactivation, particularly in heterogeneous waste feeds [254,255,256], and although specific systems (e.g., ZnCl_2_, NH_4_Y) offer marginal benefits, further research is needed on catalyst regeneration and integration with downstream refining [256,257].

The catalytic pyrolysis of LDPE shows similar improvements, with distinct product evolution compared to HDPE. Acidic zeolites, especially ZSM-5, show high selectivity toward light aromatics such as benzene, toluene, and xylenes. Under optimized low-temperature operation, ZSM-5 has achieved 50.6 wt% aromatic yield at 280 °C for 1 h with 90.9% selectivity, attributed to micropore confinement and in situ hydrogen transfer [258]. HZSM-5 enhances olefins and aromatics, whereas HUSY favors paraffins in the C_4_–C_8_ range [244,259]. Zeolite Beta, when engineered to hierarchical porosity with tuned acidity, has achieved selectivities as high as 88.7% toward lubricating base oil [260], while HY zeolites promote volatile paraffins through disproportionation mechanisms [259]. A key limitation is deactivation by coke; HZSM-5 gradually deactivates until stabilizing after several cycles [261], while LDPE generates more pronounced fouling in HUSY than HDPE [253]. Catalyst selection is a key determinant of both the kinetic and product landscape during polymer pyrolysis. Mesoporous materials such as MCM-41 and SBA-15 are widely used to improve mass transport and facilitate access of large polymer fragments to catalytic sites. MCM-41, noted for its large pore sizes and moderate acidity, enhances oil yields and reduces unwanted secondary cracking [262], while SBA-15, especially when functionalized with sulfated zirconia, delivers a high density of acid sites that promote efficient cracking, even under milder conditions [263]. Hierarchical Beta zeolites further optimize aromatic product yields by combining enhanced diffusion pathways with strong acidity, allowing for selective BTX formation [150,264].

Transition metal oxides, including alumina and silica-alumina, serve a dual role by lowering activation energies and enabling catalytic decomposition at reduced thermal loads. The use of silica-alumina, for example, substantially decreases the activation energy required for pyrolysis, from typical uncatalyzed values around 117.2 kJ mol^−1^ to 97.3 kJ mol^−1^, steering the process away from high-molecular-weight waxes and toward more valuable paraffinic and olefinic hydrocarbons. Among zeolitic catalysts, HZSM-5 stands out for its capacity to catalyze the direct conversion of primary degradation products into aromatic hydrocarbons. Its unique structure promotes BTX formation while suppressing heavy residue and wax formation. The synergistic effect of combining HZSM-5 with mesoporous or oxide catalysts can be harnessed for both lower activation energies and tailored product distributions, underscoring the importance of catalyst formulation in optimizing chemical recycling outcomes [265].

By rationally selecting and combining catalyst types—balancing pore structure, acidity, and redox properties—it is possible to direct the pyrolysis of waste plastics toward higher-value fuels and chemicals while improving energy efficiency and operational flexibility.

Carbon-based catalysts, including activated carbons and nanotubes, enable chain adsorption and breakdown, with selectivity tuned by surface functionalities [266]. Mechanistic investigations underscore carbocation intermediates as central, with ZSM-5 favoring dehydroaromatization and HY favoring iso-paraffins [258,267]. In practice, MCM-41 maximizes oil yields (78.4% at 650 °C), while ZSM-5 produces high aromatic content (65.9% at 500 °C) [262]. Silica-alumina provides paraffinic, low-aromatic liquids ideal for clean fuels [266]. Natural zeolites such as clinoptilolite can accelerate decomposition and alter selectivity, though reuse is hindered by coke [268]. Across studies, acidity, pore topology, and catalyst stability emerge as the key determinants of LDPE catalytic pyrolysis efficiency [11,253,269].

For PP, product distributions are strongly shaped by catalyst acidity, structure, and pore dimensions. HZSM-5 enhances the formation of C_3_–C_12_ hydrocarbons and aromatics such as benzene, toluene, and xylene through its microporous and strongly Brønsted acidic nature, but generally reduces wax yields compared to thermal pyrolysis [270,271]. Other zeolites, including HZSM-11 and Y, sometimes combined with metal–organic frameworks (MOFs) like MIL-53 (Cu), further increase aromatic and light-oil selectivity [246,272]. Low-cost natural zeolites (e.g., clinoptilolite) increase paraffinic liquid fractions, with milder acidity and porosity preserving longer hydrocarbons [273,274]. MOF’s such as UiO-66, rich in Lewis-acidic zirconia sites, enable selective C–C cleavage while minimizing residue and steering products toward aliphatics rather than aromatics [275]. Metal-doped systems add further flexibility: Ni–Cr supported on natural zeolites boosts gasoline-range hydrocarbons near 450 °C through combined acid and hydrogen transfer/dehydrogenation functions, while cobalt oxides reduce activation energy and yield fuel-grade oils under milder conditions (~430 °C) [276]. Silica-alumina has achieved oil yields up to 91 wt% at 500 °C, outperforming clays such as kaolin or bentonite. Spent Fluid Catalytic Cracking (FCC) catalysts are widely available and cost-effective, but tend to produce more gaseous products, limiting wax recovery [277]. Overall, zeolites consistently favor aromatic pathways, while weaker acidity and larger pore catalysts, including MOFs, are better suited for retaining paraffinic waxes under inert or vacuum conditions [278,279].

PVC pyrolysis differs fundamentally, involving initial dehydrochlorination (270–320 °C) to polyenes, followed by secondary scission, aromatization, or char formation [232,233,237,238]. Catalysts serve dual purposes: lowering onset temperatures and managing HCl. Metal chlorides such as ZnCl_2_ and BaCl_2_ accelerate dehydrochlorination, leading to early HCl and benzene release below 350 °C and shifting product distributions toward volatiles, especially under hydrogen co-feeding at 400 °C [17]. However, these may also stabilize intermediates, increasing char via metal–organic complexes. Metal hydroxides and oxides such as Ca(OH)_2_, CaO, Mg(OH)_2_, and MgO mainly act as HCl traps, reacting to form stable chlorides and producing a cleaner gas phase. Calcium systems outperform magnesium, and hydroxides outperform oxides in HCl uptake [280,281]. Transition metal oxides (MoO_3_, CuO, Fe_2_O_3_, Co_3_O_4_) alter degradation trajectories: MoO_3_ promotes char and reduces aromatics [282]; CuO favors aliphatic hydrocarbons via intermolecular crosslinking; Fe_2_O_3_ and ZnO promote deeper cracking, lowering liquids and enriching gases [281,283,284]. These behaviors reflect Lewis acidity and redox functions in stabilizing polyene intermediates. TiO_2_, ZnO, and SnO_2_ modulate gas compositions, with TiO_2_ enhancing aromatics (chlorobenzenes, naphthalenes) and ZnO/SnO_2_ suppressing aromatics in favor of lighter aliphatic gases at 400–500 °C [281]. More recent work has applied ionic liquids such as [P_4444_][Cl], achieving up to 98% dehydrochlorination at 80–180 °C in 60 min through anion-assisted stabilization of elimination intermediates [285]. Alkaline additives such as CaO and K_2_CO_3_ also capture HCl and steer degradation: CaO has reduced HCl emissions below 20% at 1:1.6 additive/PVC ratios, while K_2_CO_3_ enhances both neutralization and gas-phase yields [283]. Thus, catalytic design in PVC pyrolysis is a balance between early HCl removal, light gas maximization, char suppression, and minimization of toxic aromatics, typically optimized at 300–500 °C.

Together, these studies confirm that catalyst selection—acidity, pore structure, redox potential, and regeneration possibilities—exerts decisive influence on product selectivity and environmental implications in plastic pyrolysis. Strong Brønsted acidity and micropores favor olefin and aromatic products; mesoporous or hierarchical structures extend diffusion and enhance paraffinic and oil yields; metal functions enable hydrogen transfer, dehydrogenation, or selective dechlorination; and specific additives in PVC serve crucial roles in HCl management to assure HCl-free pyrolysis gases. Persistent bottlenecks remain in coke formation, feed impurities, and catalyst regeneration, but advances in hierarchical structures, two-stage processing, and ionic liquid dechlorination strategies point toward increasingly selective and sustainable catalytic pyrolysis.

#### 4.1.2. Initiator Effects for HDPE, LDPE, PP and PVC Pyrolysis

The use of radical and thermal initiators represents a central strategy in controlling polymer pyrolysis under vacuum conditions, where oxygen is excluded and reaction pathways are dominated by radical chemistry [11]. Initiators not only lower the degradation onset temperature but also reduce activation energies and steer selectivity toward valuable products, thereby enhancing the efficiency and scalability of vacuum pyrolysis [286].

For HDPE, pyrolysis under inert conditions usually commences around 400–500 °C, with an activation energy of about 227 kJ mol^−1^. Radical initiators reduce this threshold substantially. Peroxides such as dicumyl peroxide (DCP) or cumene hydroperoxide decompose above 300 °C to form alkoxy radicals, which abstract hydrogens from the polymer chain and initiate β-scission. More remarkable is the action of 2-ethylhexyl nitrate (2-EHN), which decomposes via O–N bond homolysis around 150 °C under oxygen-free conditions, yielding an alkoxy radical (RO·) and a nitrogen dioxide radical (NO_2_·). The RO· initiates hydrogen abstraction from the HDPE backbone, while NO_2_· further propagates radical formation or undergoes recombination to N_2_O_4_. This process provides an efficient radical source without requiring molecular oxygen, thereby reducing the energy demand of the pyrolytic system [256]. The presence and choice of radical initiators powerfully influence the kinetics and product distributions of polymer pyrolysis. Initiators lower the activation energy required for bond cleavage, accelerating the onset of depolymerization and promoting more extensive fragmentation of polymer chains. Kinetic studies show that activation energies can be reduced to approximately 122 kJ mol^−1^ when initiators are present, with notable reductions in overall enthalpy and improved product yields [287,288,289,290]. This effect is especially pronounced for low-density polyethylene (LDPE), which degrades more readily due to its branched, less crystalline structure. Flash vacuum pyrolysis experiments demonstrate that initiators enhance recovery of light olefins, often increasing ethylene content to more than 60% of the gas fraction and boosting monomer recovery. The combination of initiators with solid catalysts yields a synergistic effect: for example, co-feeding initiators with catalysts such as silica–alumina or ZnO further reduces the activation energy and increases selectivity towards gasoline- and diesel-range hydrocarbons [291,292,293].

By carefully selecting and optimizing initiators, it is possible to precisely tailor pyrolysis pathways, maximize targeted product yields, and improve the efficiency of plastic recycling processes. Oil yields of up to 80% have been reported when radical initiators are combined with mesoporous or acidic catalysts, showing the promising potential for LDPE valorization [294,295,296,297].

The pyrolysis of PP is particularly sensitive to radical initiators because of its tertiary carbon centres, which are prone to chain scission. Conventional peroxides efficiently accelerate degradation, with tetra-functional peroxides outperforming di-functional ones due to their higher radical output. Nitroxide-mediated systems (NOR) provide more controlled reductions in molecular weight, limiting runaway fragmentation and preserving tunable rheological properties. Organosulfur compounds act differently: instead of directly producing radicals, they lower the activation energy (~146 kJ mol^−1^) by stabilizing transition states, making chain scission more accessible. Organo-Sn and macromolecular initiators, such as polyglycidyl methacrylate (PGMA), contribute through additional radical release pathways but raise toxicity or stability concerns [247,298,299,300]. Synergistic use of initiators with molecular sieves or cobalt oxides reduces activation barriers to as low as ~64 kJ mol^−1^, shifting selectivity toward liquid fuels, while Ziegler–Natta catalysts, traditionally employed in polymer synthesis, can be repurposed to promote radical depolymerization at reduced temperatures [276]. Overall, radical initiators allow PP pyrolysis to be tuned between light olefin/BTX production and heavier wax/oil fractions depending on initiator chemistry and operating temperature.

The role of initiators in PVC degradation is especially critical because of the polymer’s two-step breakdown: initial dehydrochlorination at 270–320 °C, followed by polyene scission. In vacuum conditions, initiators such as ZnCl_2_ and BaCl_2_ accelerate dehydrochlorination by lowering the onset to below 350 °C, facilitating early HCl release and reducing polyene buildup [17]. Alkaline oxides and carbonates, particularly CaO and K_2_CO_3_, act as both acid scavengers and initiators, trapping up to 80% of released HCl and thereby preventing chlorine contamination of pyrolysis oils [283]. Zeolitic systems like H-ZSM-5 provide dual functionality: they initiate dehydrochlorination via their strong acidity and simultaneously convert intermediates into olefinic gases, reducing chlorine residues to as low as 20 ppm in secondary oil products [301]. Transition metal oxides, including Fe_2_O_3_ and ZnO, further alter PVC pyrolysis pathways through redox and adsorption effects, shifting selectivity toward light aliphatic gases and suppressing the formation of toxic aromatics. Recent innovations have explored ionic liquids (e.g., [P_4444_][Cl]) as initiators, achieving nearly complete dehydrochlorination at temperatures as low as 80–180 °C, thereby opening low-temperature routes for PVC valorization [285].

Taken together, initiators significantly extend the operational flexibility of vacuum pyrolysis. They reduce energy input, accelerate degradation kinetics, and steer selectivity toward fuels or specialty chemicals, while also mitigating undesirable byproducts such as waxes or chlorinated aromatics. When combined with catalysts, initiators enable finely tuned, lower-temperature processes that maximize fuel quality and minimize environmental impacts. Their chemistry is thus central to advancing vacuum pyrolysis into a scalable solution for circular plastic waste management. It is always mandatory to consider recyclability and environmental impacts.

#### 4.1.3. Termination Strategies for HDPE, LDPE, PP and PVC Pyrolysis

Termination strategies in the pyrolysis of HDPE aim to inhibit uncontrolled chain scission and radical propagation, thus enhancing selectivity toward desired liquid hydrocarbons and reducing the formation of low-molecular-weight byproducts [173]. One of the most widely studied approaches involves the use of butylated hydroxytoluene (BHT), a radical scavenger. When immobilized on hydrophobic silica fillers, BHT exhibits improved radical-scavenging activity by reducing its volatility and enhancing its interaction with the polymer matrix. This results in significantly lower yields of volatile products and a shift toward higher-molecular-weight hydrocarbons, while hydrophilic silica–BHT systems show diminished effectiveness due to weaker retention and lower scavenging activity [173]. An alternative radical-trapping approach uses fullerene (C_60_), which stabilizes carbon-centered radicals formed during HDPE decomposition. Its radical scavenging effect increases the activation energy required for degradation, thereby suppressing early-stage decomposition. This suppression slows the degradation rate under inert atmospheres and results in a greater proportion of stable, liquid-phase hydrocarbons while minimizing gaseous products [302].

Catalyst-based termination methods also offer effective control over degradation. Microporous zeolites such as HZSM-5, HY, and USY provide Brønsted acid sites that facilitate targeted cracking and secondary transformations, including isomerization and aromatization [303,304]. Their pore architecture enables shape-selective conversion, leading to an increase in liquid products rich in olefins and aromatics, with HZSM-5 favoring C_5_–C_8_ olefins and HY enhancing aromatic hydrocarbon yield, sometimes up to 50% of the liquid fraction [305]. Spent FCC catalysts represent a cost-effective and industrially scalable solution. These catalysts significantly lower the degradation temperature and increase the rate of formation of low-boiling, olefinic liquids. When applied to mixed polyolefin streams, they improve product uniformity and decrease the molecular weight distribution of the resulting liquids, aligning their composition more closely with gasoline-range hydrocarbons [306,307]. Collectively, termination modulation in HDPE pyrolysis through additives like BHT and fullerene, or via catalytic systems such as zeolites and FCC catalysts, enables suppression of undesired fragmentation pathways and enhances product selectivity [173].

In the case of LDPE, termination strategies play a crucial role in maintaining liquid yields and avoiding excessive scission into light hydrocarbons [273]. Catalytic control is again important, with natural zeolites such as clinoptilolite and Philippine natural zeolite providing cost-effective alternatives that reduce uncondensed gases while improving liquid oil recovery [273]. Rapid quenching of pyrolysis vapors also provides a physical termination mechanism, stabilizing liquid products and suppressing secondary radical-driven fragmentation into smaller volatile molecules [178]. The reusability and regeneration of catalysts, such as HZSM-5, restored at 700 °C in inert atmospheres, further support long-term stability in termination efficiency and minimize coke deposition that otherwise alters product selectivity [270]. These combined strategies for LDPE highlight the importance of both chemical and physical termination in maintaining process control.

Controlling termination in the pyrolysis of PP is particularly important to preserve long-chain hydrocarbons such as waxes and mid-distillate fuels, since uncontrolled propagation often leads to over-cracking into lighter gaseous fractions [308,309]. Thermal regulation is one of the most effective strategies, with operations in the 450–550 °C range enabling a balance between conversion efficiency and selectivity. For instance, pyrolysis at 500 °C yielded a stable liquid fraction of ~76% within 30 min, underscoring the benefit of moderate conditions [310]. Catalytic intervention further shapes the termination process, with acidic zeolites promoting gasoline-range hydrocarbons (C_5_–C_12_). At the same time, kaolin, activated carbon, and iron-modified catalysts enhance mid-range hydrocarbon selectivity and reduce oxygenated byproducts [32,311]. Optimization of operating variables, such as nitrogen flow rate and feed rate, using statistical design methods has yielded fuel oil fractions above 89%, while minimizing gas formation and suppressing excessive secondary cracking [309,312]. Rapid quenching is also widely recognized as a practical physical termination step, particularly when integrated with catalytic systems [313]. Catalyst regeneration ensures consistent activity across multiple cycles, enabling long-term process stability and controlled termination [314]. Collectively, these strategies underline the multifaceted approaches available for PP termination under inert pyrolysis conditions.

For PVC, termination strategies aim to suppress degradation beyond dehydrochlorination, thereby preventing char formation and improving the recovery of volatile hydrocarbons [283]. Since PVC degradation proceeds via initial HCl release at ~270–320 °C followed by polyene formation and eventual cross-linking, termination focuses on stabilizing these intermediates or halting their progression. Rapid cooling under vacuum effectively quenches intermediates and suppresses polyene cross-linking. A two-step pyrolysis protocol has also proven effective, where dehydrochlorination at ~300–350 °C is followed by high-temperature backbone cracking, decoupling chlorine elimination from subsequent degradation [315]. Hydrogen co-feeding provides radical termination by stabilizing polyene fragments and limiting aromatic or char pathways [17]. Alkaline hydroxides such as Ca(OH)_2_ and Mg(OH)_2_ neutralize HCl and disrupt radical propagation, further suppressing polyene formation [280]. Stabilizers, including organotin compounds and dichlorotin dioxine, delay dehydrochlorination, while ionic liquids facilitate both HCl removal and hydrogenation of intermediates [313]. Advanced additives such as alkaline earth stearates raise the activation energy for dehydrochlorination, delay degradation onset, and synergistically neutralize HCl when combined with lead stearate. Mechanistically, these strategies target the interruption of free radical propagation through either chemical scavenging or physical quenching, enabling selective recovery of volatile, non-chlorinated products [284].

## 5. Conclusions

Polymerization mechanisms were included because both polymerization and thermal degradation proceed via radical-mediated chain reactions, and the chain architecture created during synthesis (branching, defects, and stereoregularity) governs where radicals form and how chain reactions could propagate during end-of-life degradation, thereby shaping kinetics and product distributions. In the European context, this approach aligns with recent legislative milestones, circular-economy imperatives, and rapidly evolving standards for the handling and recycling of plastics.

Linear HDPE, branched LDPE, stereoregular PP with dense tertiary centers, and PVC with backbone C–Cl functionality present fundamentally different degradation routes before any reactor variable is selected. Recognizing the causality between polymerization, structure, and depolymerization enables chemical engineers and European process designers to replace empirical parameter sweeps with mechanism-anchored design of yields—whether the objective is light olefins and BTX aromatics for integration into the EU petrochemical sector, paraffinic oils and waxes for sustainable materials and fuels, or clean combustible gases for energy recovery and CHP in Europe.

For polyolefins, random scission, β-scission, hydrogen abstraction, and backbiting establish the baseline partitioning between gases and condensables. Catalytic topology and acidity then translate that intrinsic chemistry into selective outcomes: strong Brønsted acidity in shape-selective micropores compresses distributions toward C_2_–C_4_ olefins and BTX aromatics, whereas weaker acidity and meso- or hierarchical porosity preserve longer chains and favor paraffinic oils and waxes—product streams valued under EU recovery and recycling targets. Process variables map directly onto these mechanisms: temperature and heating rate set the onset and depth of primary cracking; vapor residence time and pressure regulate the extent of secondary scission, oligomerization, and aromatization; and rapid quench kinetically traps liquids before over-cracking. Initiators lower activation thresholds and tune radical populations, reducing energy input and expanding the operable window, an essential aspect for energy-lean EU recycling. Diffusion management is coequal with acidity for stability: hierarchical frameworks, external surface passivation, and judicious Al siting mitigate pore mouth blockage, slow coke nucleation, and sustain selectivity at conversion.

PVC imposes a flowsheet constraint that further underscores the value of the coupled perspective. Controlled dehydrochlorination must precede residue cracking to suppress autocatalysis, protect metallurgy, and prevent chlorine carryover, all issues central to European regulations on emissions and product safety. Continuous HCl removal or in situ scavenging, together with basic/redox co-catalysts or ionic-liquid strategies, lowers the dechlorination temperature, limits polyene cyclization and condensation, and yields cleaner gas and liquid fractions. Only after halogen management is secured should severity and residence time be applied to upgrade the dechlorinated residue, with short contact and rapid quench again decisive for suppressing aromatization and char.

Within the European framework, process design thus becomes a targeted exercise in matching feed microstructure to catalyst function and transport, and in selecting operating envelopes that deliberately pivot among off-gas, light olefins/aromatics, and paraffinic liquids. The concept of influencing pyrolysis behavior already at polymer preparation stage is presented as a high value research direction, but quantitative confirmation requires focused comparative experiments because literature datasets vary widely in temperature program, residence time, reactor configuration, additives, and contamination levels. The practical implications are clear, particularly for Europe: (i) can inform yield steering through acidity and topology selection aligned with polymer architecture and regional recycling goals, while recognizing that quantitative validation requires focused studies due to large variation in published reactor conditions and feed heterogeneity; (ii) lower specific energy demand by combining initiators with appropriate catalysts, supporting Europe’s climate and efficiency objectives; (iii) longer time-on-stream by engineering diffusion pathways that defer deactivation and support sustainable operations; and (iv) for PVC, integrated halogen capture that decouples dechlorination from backbone scission and complies with EU safety standards.

Thus, directly coupling polymerization-derived structure with depolymerization mechanisms transforms heterogeneous plastic waste from a liability into a tunable feedstock for European circularity. This strategy enables scalable, selective, and energy-lean routes that align product quality, plant operability, and the ambitious circular-carbon and sustainability objectives defined by the European Union.

## Figures and Tables

**Table 1:** Representative polymerization-derived microstructural parameters for major commodity polymers.

Polymer	Polymerization Method	Branching Degree (per 1000 C Atoms)	Tacticity/Stereoregularity	M_w_/M_n_	Refs.
HDPE	Ziegler–Natta/Metallocene coordination	<0.1–2	N/A (linear)	3–12	[128,130,136]
LDPE	Radical (High Pressure)	6–12	N/A (branched)	5–8	[128,137,138]
LLDPE	Ziegler–Natta/Metallocene coordination copolymerization (ethylene + α-olefin)	2–8	N/A (linear + SCB)	2–6	[139,140]
PP (isotactic)	Ziegler–Natta/Metallocene coordination	Methyl side chains	Isotactic > 90%	4–8	[132,133,141]
PVC	Radical (vinyl chloride)	Linear backbone with Cl	Syndiotactic/Atactic	2–3	[133,134,135]

## Data Availability

No new data were created or analyzed in this study. Data sharing does not apply to this article.

## Abbreviations

The following abbreviations are used in this manuscript:

PVC	Poly(vinyl chloride)
PE HD/MD	Polyethylene High and Medium Density
PE LD/LLD	Polyethylene Low and Linear Low Density
PP	Polypropylene
BTX	Benzene, Toluene and Xylenes
EU	European Union
HZSM-5	Hydrogen form Zeolite Socony Mobil-5
CHP	Combined Heat and Power
VOC	Volatile Organic Compound
TG-FTIR	Thermogravimetric—Fourier Transform Infrared Spectroscopy
HMFI	H-form MFI-type zeolite
PCM	Phase Change Materials
Et_3_Al	triethylaluminum
MAO	Methylaluminoxane
BHE	β-hydride elimination
BHT	β-hydride transfer to monomer
SBI	s-bis(indenyl)
TSA	Syn Transition State
TSC	Anti Transition State
CLDT	Chain-length-dependent termination
PLP	Pulsed Laser Polymerization
EPR	Electron Paramagnetic Resonance
MWD	Molecular Weight Distribution
SP-PLP-EPR	Single Pulse-Pulsed Laser Polymerization-Electron Paramagnetic Resonance
AIBN	Azobisisobutyronitrile
MCR	Mid-chain radical
G3(MP2)-RAD	Gaussian-3 (Møller–Plesset 2)—Radical
ONIOM	Our-N-layer Integrated Orbital and Molecular Mechanics
SCB	Short-chain Branching
GPC	Gel Permeation Chromatography
NMR	Nuclear Magnetic Resonance
DSC	Differential Scanning Calorimetry
TGA	Thermogravimetric Analysis
PAH	Polycyclic aromatic hydrocarbons
MNDO	Modified Neglect of Diatomic Overlap
AM1	Austin Model 1
DFT	Density Functional Theory
HY	Hydrogen-form Y-type Zeolite
MCM-41	Mobil Composition of Matter 41
SBA-15	Santa Barbara Amorphous-15
HUSY	Hydrogen-form Ultra-Stable Y-type Zeolite
HBEA	Hydrogen-form Beta Zeolite
MOF	Metal–Organic Framework
FCC	Fluid Catalytic Cracking
DCP	Dicumyl peroxide
2-EHN	2-ethylexyl nitrate
NOR	Nitroxide-mediated system
PGMA	Polyglycidyl methacrylate
BHT	Butylated hydroxytoluene
